# Supplementary material for: Direct observation of subunit rotation during DNA strand exchange by serine recombinases
Source: Nat Commun. 2024 Nov 29;15:10407. doi: 10.1038/s41467-024-54531-4 (PMC11607074; doi:10.1038/s41467-024-54531-4)
Supplement: Supplementary file 1 — Supplementary Information [file 41467_2024_54531_MOESM1_ESM.pdf]

Supplementary Information

Supplementary Tables

Supplementary table 1. Sequences of ssDNA oligonucleotides used to form various site I substrates for Sin Q115R and Tn3 NM resolvases.

Purpose	Sequence and labelling position (5'-3')	Fluorophore
Tn3 resolvase single-site substrate, 'Ser1', top	**B**CGTGACTCAACCGTTCGAAATATTATAAAXTATCAGACATAGTGGGGCGG	X = T-Cy5<br>B = biotin
Tn3 resolvase single-site substrate, 'Ser1', bottom	CCGCCCCACTATGTCTGATAATTTATAATAXTTCGAACGGTTGAGTCACG	X = T-Alexa 488
Tn3 resolvase single-site substrate, 'Ser2', top	GGCAAGCCAGCGTTGCGTGACTCAACCGTTCGAAATATTATAAATTATCAGACATAGTGGGA TGGTCTAGGCCGCAGCGG	-
Tn3 resolvase single-site substrate, 'Ser2', bottom	CCGCTGCGGCCTAGACCATCCCACTATGTCTGATAATTTATAATATTTCGAACGGTTGAGTC ACGCAACGCTGGCTTGCC	-
Sin resolvase single-site substrate, 'Sin2Ser1', top	CGTGACTCAACTGTGAAATTXGGGTATACCCTAATCATACATAGTGGGGCGG	X = T-Cy5
Sin resolvase single-site substrate, 'Sin2Ser1', bottom	CCGCCCCACTATGTATGATXAGGGTATACCCAAATTTCACAGTTGAGTCACG	X = T-Alexa 488
Sin resolvase single-site substrate, 'SinSer2', top	GGCAAGCCAGCGTTGCGTGACTCAACTGTGAAATTTGGGTATACCCTAATCATA CATAGTGGGATGGTCTAGGCCGCAGCGG	-
Sin resolvase single-site substrate, 'SinSer2', bottom	CCGCTGCGGCCTAGACCATCCCACTATGTATGATTAGGGTATACCCAAATTTCACAGTTGAG TCACGCAACGCTGGCTTGCC	-
Tn3 resolvase linked site I substrate 'UL25', X-arm top	**B**GGGTCCGTGACTCAACCGTTCGAAATATTATAAAXTATCAGACAGTGGGGTGG	X = T-Cy5<br>B = biotin
Tn3 resolvase linked site I substrate 'UL25', Y-arm top	CGCCCGTTCGAAATATTATAAATTATCAGACACCTCACCCCGGCCCCAACGCGTCCC	-
Tn3 resolvase linked site I substrate 'UL25', X-arm bottom	CCAGGATCCCGCCCCGCCACCCCACTGTCTGATAATTTATAATATTTCGAACGGTTGAGTCA CGGACCC	-
Tn3 resolvase linked site I substrates 'UL25', Y-arm bottom	GGGGTGAGGTGTCTGATAATTTATAATAXTTCGAACGGGCG<br>GGGGTGAGGTGTCTGATAATTTATAATAXTTCGAACGGGCG	X = T-Alexa 488 (MFD)<br>X = T-Atto 532 (TIRF)
25-thymine linker (UL25 and SUL25)	CGGGGCGGGATCCTGGTTTTTTTTTTTTTTTTTTTTTTTTTGGGACGCGTTGGGGCC	-
Sin resolvase linked site I substrate 'SUL25', X-arm top	**B**GGGTCCGTGACTCAACTGTGAAATTXGGGTATACCCTAATCATACAGTGGGGTGG	X = T-Cy5<br>B = biotin
Sin resolvase linked site I substrate 'SUL25', X-arm bottom	CCAGGATCCCGCCCCGCCACCCCACTGTATGATTAGGGTATACCCAAATTTCACAGTTGAG TCACGGACCC	-
Sin resolvase linked site I substrate 'SUL25', Y-arm top	CGCCTGTGAAATTTGGGTATACCCTAATCATACACCTCACCCCGGCCCCAACGCGTCCC	-
Sin resolvase linked site I substrate 'SUL25', Y-arm bottom	GGGGTGAGGTGTATGATXAGGGTATACCCAAATTTCACAGGCG<br>GGGGTGAGGTGTATGATXAGGGTATACCCAAATTTCACAGGCG	X = T-Alexa 488 (MFD)<br>X = T-Atto 532 (TIRF)

Supplementary table 2. Summary of the effect of fluorophores on the recombination of single-site substrates ‘Ser1’ and ‘Ser 2’ by Tn3 NM.

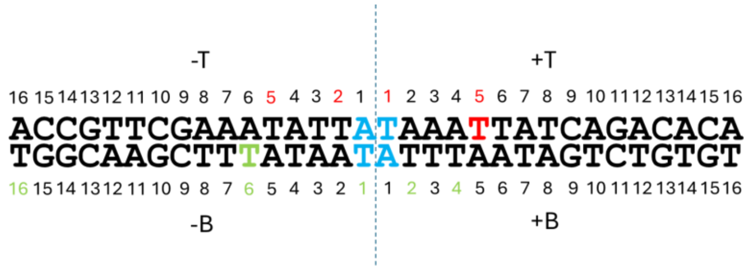

**Strand exch. detection**	-	-	+	+	+	-	-	+	+	+	-	-	+	+	+	+	+	-	-	-
**Cy5 pos. top-strand**	- 5	- 5	- 5	- 5	- 5	- 2	- 2	- 2	- 2	- 2	+ 1	+ 1	**+ 1**	+ 1	+ 1	+ 5	**+ 5**	+ 5	+ 5	+ 5
**Cy3 pos. bottom-strand**	- 16	- 6	- 1	+ 2	+ 4	- 16	- 6	- 1	+ 2	+ 4	- 16	- 6	**- 1**	+ 2	+ 4	- 16	**- 6**	- 1	+ 2	+ 4
**Recombinant**	5	5	3	5	5	2	2	0	2	2	0	0	**0**	0	0	5	**5**	0	2	3
**Nicked recombinant**	1	1	1	1	1	3	3	1	2	2	1	1	**0**	1	1	1	**1**	1	3	1
**Nicked substrate**	2	1	2	3	3	3	3	3	3	3	3	3	**5**	3	3	1	**1**	3	3	2
**DSB**	3	2	4	3	3	3	3	5	4	3	5	5	**2**	5	5	2	**2**	4	3	2

Substrates were reacted with Tn3 NM resolvase at 37 °C for one hour and the reactions were stopped by the addition of SDS loading buffer. Note that the substrates comprise ‘Ser 1’ oligos, but with fluorophores in the alternative positions specified. Protease K was added subsequently to digest the protein units of covalent complexes. Fluorophores were attached at different positions, to the right (R) or left (L) of the central dinucleotide (See Fig. 2B for R5, L6 example). To estimate the amount of recombination products, the band intensities in the gel images (example shown in Supplementary Fig.1) were compared by eye. The numbers represent relative amounts of reaction products on an arbitrary scale, with "0" for no visible detection and "5" for the highest amount found in the samples. The ‘recombinant’ values were determined from samples which had not yet been digested with protease K (i.e., with covalent linkages to Tn3 subunits) to accurately understand how the fluorophores could inhibit the rotation step. The results using fluorophore positions that were chosen for further investigation with Tn3 NM resolvase are circled.

Supplementary table 3. The relative populations of molecules undergoing various transitions during recombination, and rate constants of each transition recovered from cumulative dwell time histograms.

Single exponential analysis					Biexponential analysis							
Resolvase	Transition	Relative pop	kj (s^{-1})	kjj' (s^{-1})	τ_1 (s)	A_1 (%)	kj (1) (s^{-1})	τ_2 (s)	A_2 (%)	kj(2) (s^{-1})	k_{av} j	k_{av} jj'
Sin Q115R	lnr to cnr	0.09	0.65 (0.18)	0.18 (0.02)	0.30 (0.01)	62.9 (5.2)	3.33 (0.12)	1.87 (0.49)	37.1 (5.2)	0.53 (0.14)	1.13 (0.21)	**0.32 (0.06)**
Sin Q115R	lnr to cr	0.17	0.65 (0.18)	0.32 (0.03)	0.30 (0.01)	62.9 (5.2)	3.33 (0.12)	1.87 (0.49)	37.1 (5.2)	0.53 (0.14)	1.13 (0.21)	**0.56 (0.10)**
Sin Q115R	lnr to lr	0.08	0.65 (0.18)	0.15 (0.01)	0.30 (0.01)	62.9 (5.2)	3.33 (0.12)	1.87 (0.49)	37.1 (5.2)	0.53 (0.14)	1.13 (0.21)	**0.26 (0.05)**
Sin Q115R	cnr to lnr	0.07	1.28 (0.06)	0.52 (0.07)	0.29 (0.02)	44.2 (6.4)	3.45 (0.19)	1.00 (0.26)	55.8 (6.4)	1.00 (0.26)	1.46 (0.16)	**0.59 (0.06)**
Sin Q115R	cnr to cr	0.10	1.28 (0.06)	0.75 (0.11)	0.29 (0.02)	44.2 (6.4)	3.45 (0.19)	1.00 (0.26)	55.8 (6.4)	1.00 (0.26)	1.46 (0.16)	**0.86 (0.09)**
Sin Q115R	cnr to lr	0.01	1.28 (0.06)	0.01 (0.01)	0.29 (0.02)	44.2 (6.4)	3.45 (0.19)	1.00 (0.26)	55.8 (6.4)	1.00 (0.26)	1.46 (0.16)	**0.01 (0.001)**
Sin Q115R	cr to lnr	0.20	1.40 (0.21)	0.79 (0.11)	0.17 (0.01)	59.0 (1.5)	5.88 (1.16)	0.88 (0.19)	41.0 (1.5)	1.14 (0.24)	2.02 (0.08)	**1.23 (0.05)**
Sin Q115R	cr to cnr	0.08	1.40 (0.21	0.31 (0.05)	0.17 (0.03)	59.0 (1.5)	5.88 (1.16)	0.88 (0.19)	41.0 (1.5)	1.14 (0.24)	2.02 (0.08)	**0.48 (0.02)**
Sin Q115R	cr to lr	0.07	1.40 (0.21)	0.29 (0.04)	0.17 (0.03)	59.0 (1.5)	5.88 (1.16)	0.88 (0.19)	41.0 (1.5)	1.14 (0.24)	2.02 (0.08)	**0.45 (0.02)**
Sin Q115R	lr to lnr	0.07	1.38 (0.50)	0.60 (0.22)	0.18 (0.03)	69.2 (4.3)	5.56 (1.05)	1.20 (0.37)	30.8 (4.3)	0.83 (0.25)	0.83 (0.13)	**0.89 (0.06)**
Sin Q115R	lr to cnr	0.01	1.38 (0.50)	0.01 (0.01)	0.18 (0.03)	69.2 (4.3)	5.56 (1.05)	1.20 (0.37)	30.8 (4.3)	0.83 (0.25)	0.83 (0.13)	**0.01 (0.001)**
Sin Q115R	lr to cr	0.09	1.38 (0.50)	0.76 (0.28)	0.18 (0.03)	69.2 (4.3)	5.56 (1.05)	1.20 (0.37)	30.8 (4.3)	0.83 (0.25)	0.83 (0.13)	**1.13 (0.07)**
Sin Q115R (Mg)	lnr to cnr	0.14	0.46 (0.09)	0.13 (0.01)	0.24 (0.03)	68.3 (5.4)	4.17 (0.33)	3.28 (0.13)	31.7 (5.4)	0.30 (0.01)	3.07 (0.18)	**0.24 (0.05)**
Sin Q115R (Mg)	lnr to cr	0.16	0.46 (0.09)	0.15 (0.01)	0.24 (0.02)	68.3 (5.4)	4.17 (0.33)	3.28 (0.13)	31.7 (5.4)	0.30 (0.01)	3.07 (0.18)	**0.27 (0.06)**
Sin Q115R (Mg)	lnr to lr	0.05	0.46 (0.09)	0.05 (0.01)	0.24 (0.02)	68.3 (5.4)	4.17 (0.33)	3.28 (0.13)	31.7 (5.4)	0.30 (0.01)	3.07 (0.18)	**0.09 (0.02)**
Sin Q115R (Mg)	cnr to lnr	0.14	1.19 (0.03)	0.63 (0.05)	0.08 (0.02)	63.8 (5.9)	12.5 (0.58)	1.03 (0.25)	36.2 (5.9)	0.97 (0.24)	3.18 (0.11)	**1.26 (0.06)**
Sin Q115R (Mg)	cnr to cr	0.08	1.19 (0.03)	0.38 (0.03)	0.08 (0.01)	63.8 (5.9)	12.5 (0.58)	1.03 (0.25)	36.2 (5.9)	0.97 (0.24)	3.18 (0.11)	**0.75 (0.04)**
Sin Q115R (Mg)	cnr to lr	0.04	1.19 (0.03)	0.17 (0.01)	0.08 (0.01)	63.8 (5.9)	12.5 (0.58)	1.03 (0.25)	36.2 (5.9)	0.97 (0.24)	3.18 (0.11)	**0.35 (0.02)**
Sin Q115R (Mg)	cr to lnr	0.16	1.48 (0.16)	0.87 (0.10)	0.12 (0.01)	74.7 (5.0)	8.33 (0.27)	0.89 (0.36)	25.3 (5.0)	1.12 (0.45)	2.36 (0.10)	**1.86 (0.06)**
Sin Q115R (Mg)	cr to cnr	0.08	1.48 (0.16)	0.45 (0.05)	0.12 (0.01)	74.7 (5.0)	8.33 (0.27)	0.89 (0.36)	25.3 (5.0)	1.12 (0.45)	2.36 (0.10)	**0.97 (0.03)**
Sin Q115R (Mg)	cr to lr	0.03	1.48 (0.16)	0.16 (0.02)	0.12 (0.01)	74.7 (5.0)	8.33 (0.27)	0.89 (0.36)	25.3 (5.0)	1.12 (0.45)	2.36 (0.10)	**0.34 (0.01)**
Sin Q115R (Mg)	lr to lnr	0.05	1.50 (0.26)	0.61 (0.11)	0.10 (0.01)	71.8 (6.0)	10.0 (0.45)	0.90 (0.43)	28.2 (6.0)	1.11 (0.53)	3.07 (0.13)	**1.24 (0.05)**
Sin Q115R (Mg)	lr to cnr	0.04	1.50 (0.26)	0.51 (0.09)	0.10 (0.01)	71.8 (6.0)	10.0 (0.45)	0.90 (0.43)	28.2 (6.0)	1.11 (0.53)	3.07 (0.13)	**1.04 (0.05)**
Sin Q115R (Mg)	lr to cr	0.03	1.50 (0.26)	0.38 (0.07)	0.10 (0.01)	71.8 (6.0)	10.0 (0.45)	0.90 (0.43)	28.2 (6.0)	1.11 (0.53)	3.07 (0.13)	**0.78 (0.03)**
Sin Q115R (RT)	lnr to cnr	0.26	0.42 (0.03)	0.29 (0.02)	0.34 (0.01)	67.6 (4.0)	2.98 (0.04)	3.63 (0.28)	32.4 (4.0)	0.28 (0.02)	0.71 (0.17)	**0.49 (0.12)**
Sin Q115R (RT)	lnr to cr	0.10	0.42 (0.03)	0.11 (0.01)	0.34 (0.01)	67.6 (4.0)	2.98 (0.04)	3.63 (0.28)	32.4 (4.0)	0.28 (0.02)	0.71 (0.17)	**0.18 (0.04)**
Sin Q115R (RT)	lnr to lr	0.03	0.42 (0.03)	0.03 (0.01)	0.34 (0.01)	67.6 (4.0)	2.98 (0.04)	3.63 (0.28)	32.4 (4.0)	0.28 (0.02)	0.71 (0.17)	**0.05 (0.01)**
Sin Q115R (RT)	cnr to lnr	0.24	0.83 (0.06)	0.59 (0.05)	0.26 (0.01)	64.5 (4.7)	3.85 (0.08)	1.93 (0.20)	35.5 (4.7)	0.52 (0.05)	1.17 (0.12)	**0.84 (0.20)**
Sin Q115R (RT)	cnr to cr	0.07	0.83 (0.06)	0.17 (0.01)	0.26 (0.01)	64.5 (4.7)	3.85 (0.08)	1.93 (0.20)	35.5 (4.7)	0.52 (0.05)	1.17 (0.12)	**0.24 (0.06)**
Sin Q115R (RT)	cnr to lr	0.03	0.83 (0.06)	0.07 (0.01)	0.26 (0.01)	64.5 (4.7)	3.85 (0.08)	1.93 (0.20)	35.5 (4.7)	0.52 (0.05)	1.17 (0.12)	**0.09 (0.02)**
Sin Q115R (RT)	cr to lnr	0.11	0.93 (0.09)	0.50 (0.05)	0.18 (0.01)	67.1 (6.8)	5.58 (0.12)	1.79 (0.25)	32.9 (6.8)	0.56 (0.08)	1.41 (0.15)	**0.75 (0.18)**
Sin Q115R (RT)	cr to cnr	0.06	0.93 (0.09)	0.29 (0.03)	0.18 (0.01)	67.1 (6.8)	5.58 (0.12	1.79 (0.25)	32.9 (6.8)	0.56 (0.08)	1.41 (0.15)	**0.44 (0.11)**
Sin Q115R (RT)	cr to lr	0.03	0.93 (0.09)	0.14 (0.01)	0.18 (0.01)	67.1 (6.8)	5.58 (0.12	1.79 (0.25)	32.9 (6.8)	0.56 (0.08)	1.41 (0.15)	**0.21 (0.05)**
Sin Q115R (RT)	lr to lnr	0.02	1.88 (0.83)	0.52 (0.23)	0.20 (0.01)	73.6 (11.0)	4.99 (0.11)	1.92 (1.00)	26.4 (11.0)	0.52 (0.27)	1.52 (0.34)	**0.42 (0.10)**
Sin Q115R (RT)	lr to cnr	0.03	1.88 (0.83)	0.65 (0.29)	0.20 (0.01)	73.6 (11.0)	4.99 (0.11)	1.92 (1.00)	26.4 (11.0)	0.52 (0.27)	1.52 (0.34)	**0.53 (0.13)**
Sin Q115R (RT)	lr to cr	0.03	1.88 (0.83)	0.71 (0.31)	0.20 (0.01)	73.6 (11.0)	4.99 (0.11)	1.92 (1.00)	26.4 (11.0)	0.52 (0.27)	1.52 (0.34)	**0.58 (0.14)**
Tn3 NM	lnr to cnr	0.09	0.55 (0.06)	0.42 (0.05)	0.15 (0.01)	72.8 (2.4)	6.67 (0.34)	2.48 (0.12)	27.6 (2.4)	0.40 (0.02)	1.26 (0.07)	**0.95 (0.05)**
Tn3 NM	lnr to cr	0.01	0.55 (0.06)	0.05 (0.01)	0.15 (0.01)	72.8 (2.4)	6.67 (0.34)	2.48 (0.12)	27.6 (2.4)	0.40 (0.02)	1.26 (0.07)	**0.12 (0.01)**
Tn3 NM	lnr to lr	0.02	0.55 (0.06)	0.08 (0.01)	0.15 (0.01)	72.8 (2.4)	6.67 (0.34)	2.48 (0.12)	27.6 (2.4)	0.40 (0.02)	1.26 (0.07)	**0.19 (0.01)**
Tn3 NM	cnr to lnr	0.10	0.84 (0.15)	0.32 (0.06)	0.14 (0.01)	68.9 (6.2)	7.14 (0.23)	1.44 (0.04)	31.1 (6.2)	0.69 (0.02)	1.84 (0.09)	**0.69 (0.03)**
Tn3 NM	cnr to cr	0.16	0.84 (0.15)	0.52 (0.14)	0.14 (0.01)	68.9 (6.2)	7.14 (0.23)	1.44 (0.04)	31.1 (6.2)	0.69 (0.02)	1.84 (0.09)	**1.14 (0.06)**
Tn3 NM	cnr to lr	0.01	0.84 (0.15)	0.01 (0.01)	0.14 (0.01)	68.9 (6.2)	7.14 (0.23)	1.44 (0.04)	31.1 (6.2)	0.69 (0.02)	1.84 (0.09)	**0.01 (0.001)**
Tn3 NM	cr to lnr	0.03	0.60 (0.04)	0.04 (0.02)	0.23 (0.01)	57.9 (2.4)	4.35 (0.15)	2.02 (0.04)	42.1 (2.4)	0.50 (0.01)	1.02 (0.05)	**0.07 (0.01)**
Tn3 NM	cr to cnr	0.15	0.60 (0.04)	0.25(0.01)	0.23 (0.01)	57.9 (2.4)	4.35 (0.15)	2.02 (0.04)	42.1 (2.4)	0.50 (0.01)	1.02 (0.05)	**0.41 (0.02)**
Tn3 NM	cr to lr	0.20	0.60 (0.04)	0.31 (0.02)	0.23 (0.01)	57.9 (2.4)	4.35 (0.15)	2.02 (0.04)	42.1 (2.4)	0.50 (0.01)	1.02 (0.05)	**0.53 (0.03)**
Tn3 NM	lr to lnr	0.03	0.56 (0.07)	0.08 (0.01)	0.55 (0.02)	68.2 (5.4)	1.82 (0.08)	3.30 (0.18)	31.8 (5.4)	0.30 (0.02)	0.70 (0.19)	**0.10 (0.03)**
Tn3 NM	lr to cnr	0.01	0.56 (0.07)	0.01 (0.01)	0.55 (0.02)	68.2 (5.4)	1.82 (0.08)	3.30 (0.18)	31.8 (5.4)	0.30 (0.02)	0.70 (0.19)	**0.01 (0.001)**
Tn3 NM	lr to cr	0.20	0.56 (0.07)	0.48 (0.06)	0.55 (0.02)	68.2 (5.4)	1.82 (0.08)	3.30 (0.18)	31.8 (5.4)	0.30 (0.02)	0.70 (0.19)	**0.60 (0.16)**

The Sin Q115R data show the results of SUL25 recombination; the Tn3 data show the results of UL25 recombination**.** For single exponential fitting, cumulative dwell time histograms were provided after TDP clustering in MASH-FRET. The corrected rate constants ($k_{jj'}$) were determined from values extracted from single-exponential fitting of the cumulative dwell time histograms (k_j), by multiplying by the relative weight of the individual 2-D Gaussian in the mixture used to fit the TDP. The error in k (shown in parentheses) was determined from an estimate of the variability of biexponential fitting parameters across the sample by BOBA-FRET analysis[1]. Rates and errors from biexponential decays were determined similarly to that of single exponential decays. See materials and methods for calculations used to determine the average rate constant from biexponential decays (k_{av} j) and associated error.

Supplementary table 4. Effect of the linker length on the Tn3 NM resolvase-mediated recombination of linked-site substrates with varying linker lengths (UL25, UL40 and UL50).

Linker length	**25**	**25**	40	40	50	50
Time (min)	**60**	**60**	60	60	60	60
BamHI	**+**	**-**	+	-	+	-
MluI	**-**	**+**	-	+		+
% substrate	**29**	**23**	31	27	19	17
% inversion	**26**	**29**	26	19	26	15
% deletion	**10**	**15**	16	24	17	27
% intermolecular	**6**	**6**	5	6	5	6
% DSB	**30**	**27**	23	25	33	35

See Supplementary Fig. 4 for details of the gel-based recombination assay with UL25 and Tn3 NM. The T_{25} linker (bold) was chosen for the linked site substrates as the highest ratio of inversion: deletion recombinant product was obtained. Bands were digested with BamHI (columns 1, 3, 5) or MluI (columns 2, 4, 6) for 1 h, however values may differ due to incomplete restriction digest. The intensities of the bands were quantified from the processed gel images using the computer program ImageGauge (Fujifilm).

Supplementary Figures

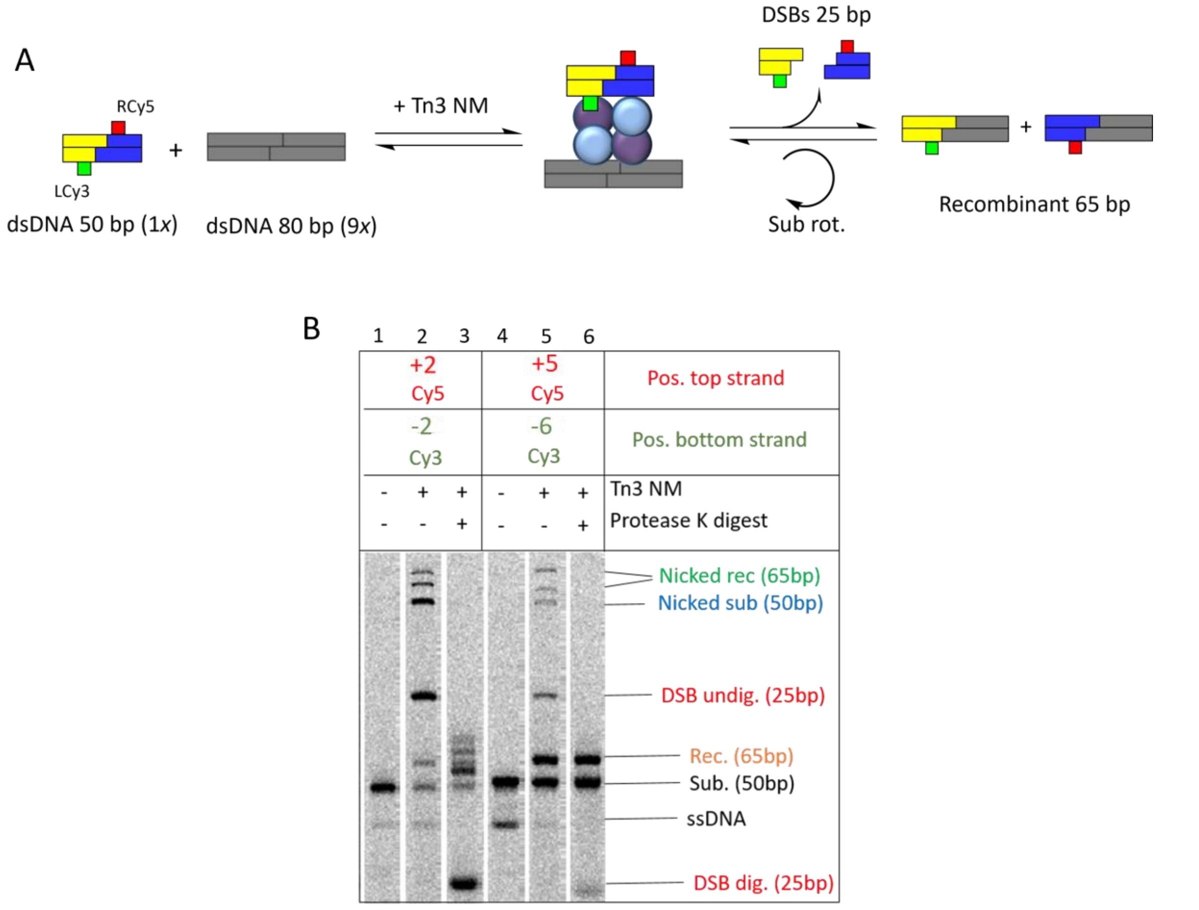

Supplementary figure 1: Effects of fluorophore position on the recombination of single-site substrates by Tn3 NM resolvase. A. Double-stranded DNA substrate (Ser1, 50 bp) containing a Cy3 at various positions to the left of site I on the bottom strand (- Cy3) and Cy5 at various positions to the right of site I on the top strand (+ Cy5) was reacted with a ninefold excess of a longer (80 bp) non-fluorescent site I duplex. Products exhibit a loss of FRET signal because the fluorophores are on separate recombinant product molecules (65 bp). **B.** Two examples are illustrated here; the site I substrate used for lanes 1-3 has Cy5 at top strand position +2 and Cy3 at bottom strand position -2 (+2T, -2B), whereas the substrate used in lanes 4-6 has Cy5 at top strand position +5 and Cy3 at bottom strand position -6 (+5T, -6B). 'Ser1' substrate positions are labelled as defined in supplementary 2. For substrate +2T -2B, recombinant formation (band labelled rec.) is low, in contrast to substrate +5T, -6B. Products of resolvase-mediated DNA strand cleavage (nicked sub, nicked rec, DSB undig.) in lanes 2 and 5 have reduced gel mobility due to an attached resolvase subunit; this is removed by protease K treatment (lanes 3 and 6). These cleavage products indicate failure to complete recombination, and the low levels with substrate +5T, -6B are a further indicator of efficient reaction. Supplementary table 2 summarizes our analysis of 20 site I substrates with varying positions of the fluorophore pair.

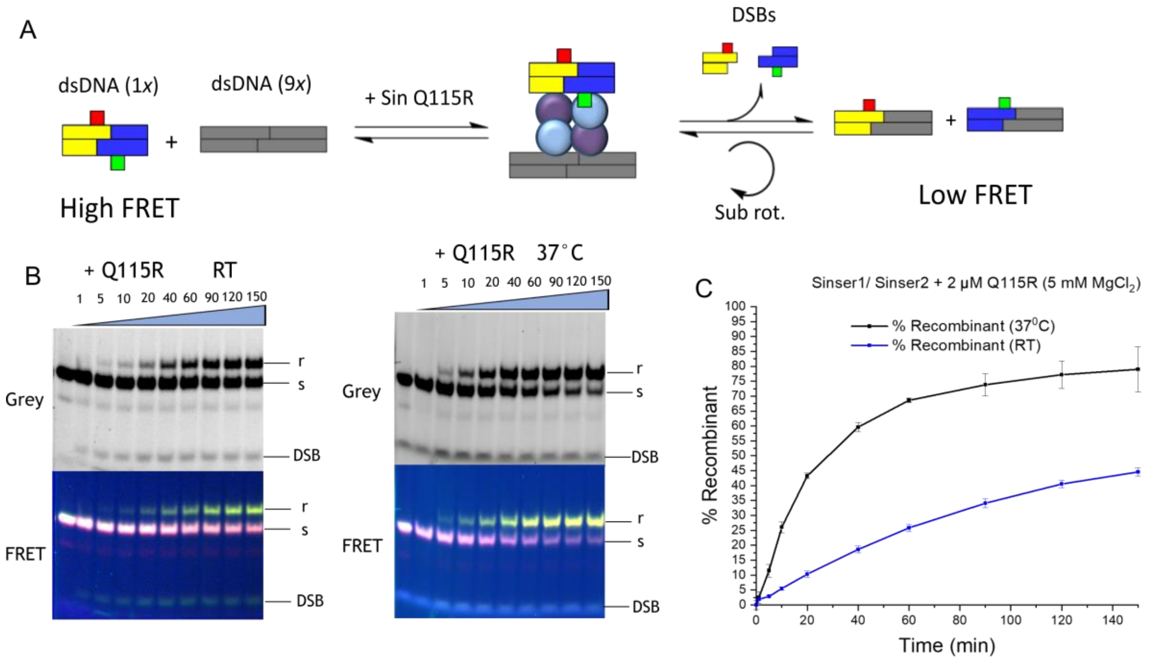

Supplementary figure 2: Time course of Sin-mediated recombination. Recombination time course assay with single site substrate SinSer2 + 2 μM Sin Q115R (reaction buffer was as defined in the Methods section, with the addition of 5 mM $MgCl_2$ and 50 μg/ml poly(dI/dC). **A**. Double-stranded DNA substrate (SinSer2, 50 bp) containing an Atto 532/ Cy5 fluorophore pair was reacted with a ninefold excess of a longer (80 bp) non-fluorescent site I duplex. Products exhibit a loss of FRET signal because the fluorophores are on separate recombinant product molecules (65 bp). **B.** SinSer2 substrate (s), recombinant (r) and double-strand break (DSB) fragments separated by electrophoresis on an 8% polyacrylamide gel containing 0.1% w/v SDS. Reactions were for 150 minutes at room temperature (left panel) and 37 °C (right panel), and were stopped at various time points by addition of a buffer containing SDS and proteinase K. The 'FRET scan' (see Methods) of the fragments is shown in colour (bottom) and greyscale (top). The pink/white bands correspond to the substrate (high FRET), whereas the green bands are recombinant, which has only the donor fluorophore (zero FRET). **C.** Plot of % recombinant against reaction time determined by quantitation of band fluorescence following gel electrophoresis as in panel B. Data points are mean values from triplicate repeats of the recombination assay.

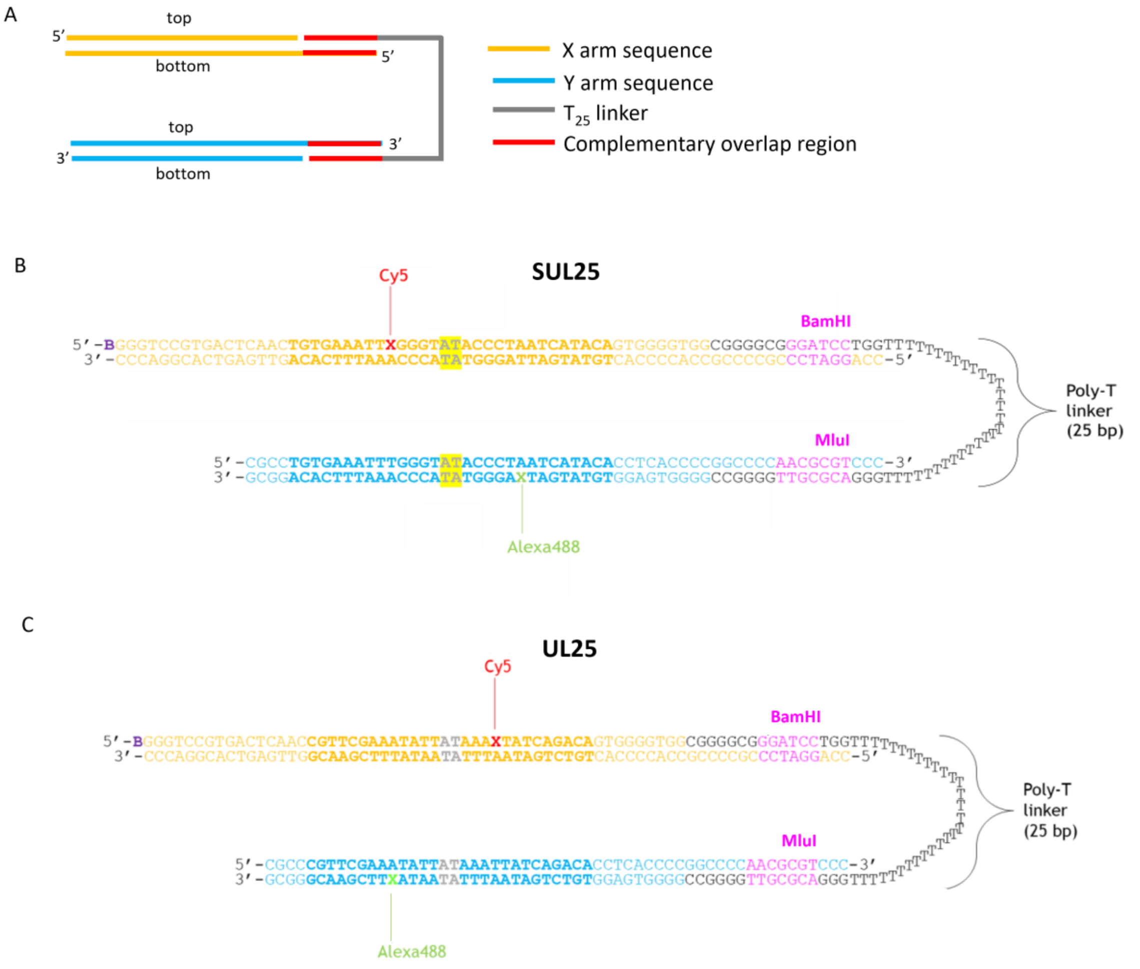

Supplementary figure 3: Design of linked site I substrates SUL25 and UL25. A. Cartoon showing how linked site substrates are assembled**.** X-arm oligos are shown in orange, Y-arm oligos in blue, T_{25} linker in grey, and the complementary overlap regions used to anneal them together are illustrated in red. **B-C.** The site I sequence for Sin or Tn3 resolvase is in bold, with the central overlap in grey. The locations of the donor (green) and acceptor (red) fluorophores are such that recombination should produce high-FRET products from a low-FRET starting configuration. The thymine linker (dark grey) is joined to X and Y arms by an overhang sequence. Binding sites for restriction endonucleases BamHI and MluI have been inserted to allow separation of recombinant product fragments on a polyacrylamide gel and are shown in magenta. There is a biotin labelled B on the X-arm top strand for surface tethering in TIRF experiments. Note that an Alexa 488/ Cy5 FRET pair (as shown) was used in MFD.

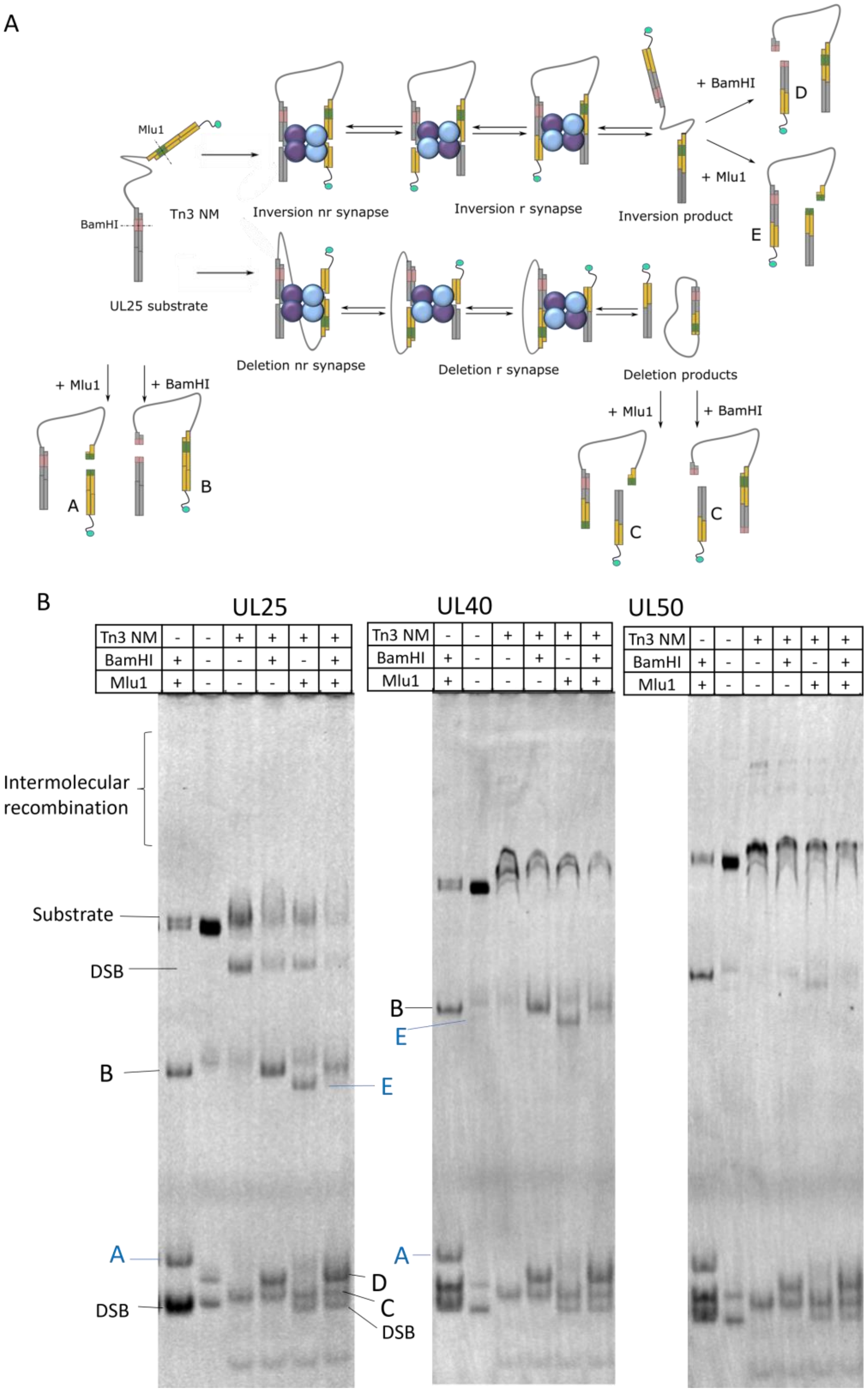

Supplementary figure 4: Recombination of linked-site substrate UL25 by Tn3 NM resolvase A. Reaction scheme of recombination with UL25 (as well as equivalent substrates UL40, UL50 with longer linkers) showing the position of the fluorescein (shown as green circle) at each stage of recombination before and after digest with BamHI or MluI. Visible fragments with a fluorescein resulting from restriction digest with MluI and BamHI of the substrate (A and B), deletion product (C) and Inversion products (D and E) are labelled. **B.** Fluorescein-labelled substrates with thymine linkers of three different lengths (T_{25}, T_{40}, and T_{50}) were treated with Tn3 NM resolvase (1.2 µM) in a buffer containing 50 mM Tris-HCl (pH 8.2), 50 µg/ml poly(dI-dC), $MgCl_2$ (10 mM) for one hour at 20 °C. The reaction products were digested with the restriction endonucleases BamHI and/or MluI followed by digestion with protease K. The DNA fragments were visualised with a fluorescence imager (See Methods).

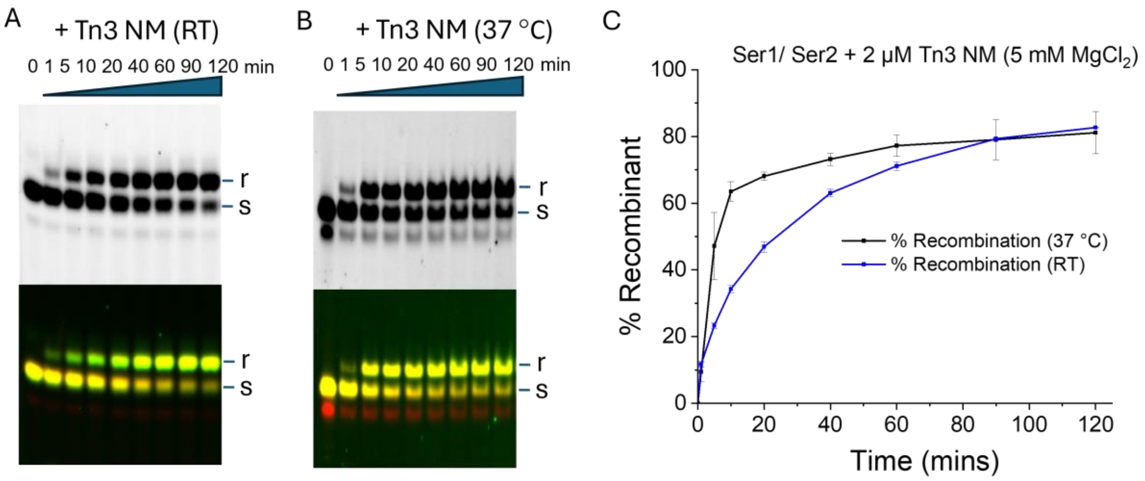

Supplementary figure 5: Time course of Tn3 NM resolvase-mediated recombination. Recombination time course assay of linear substrates with the addition of 5 mM $MgCl_2$ and 50 μg/ml poly(dI/dC). **A**. Double-stranded DNA substrate (SinSer2, 50 bp) containing a Cy3B/Cy5 fluorophore pair was reacted with a ninefold excess of a longer (80 bp) non-fluorescent site I duplex at RT. Products exhibit a loss of FRET signal because the fluorophores are on separate recombinant product molecules (65 bp). **B.** The same experiment as in A was conducted at 37 °C. **C.** Plot of % recombinant against reaction time determined by quantitation of band fluorescence following gel electrophoresis as in panel B. Data points are mean values from triplicate repeats of the recombination assay.

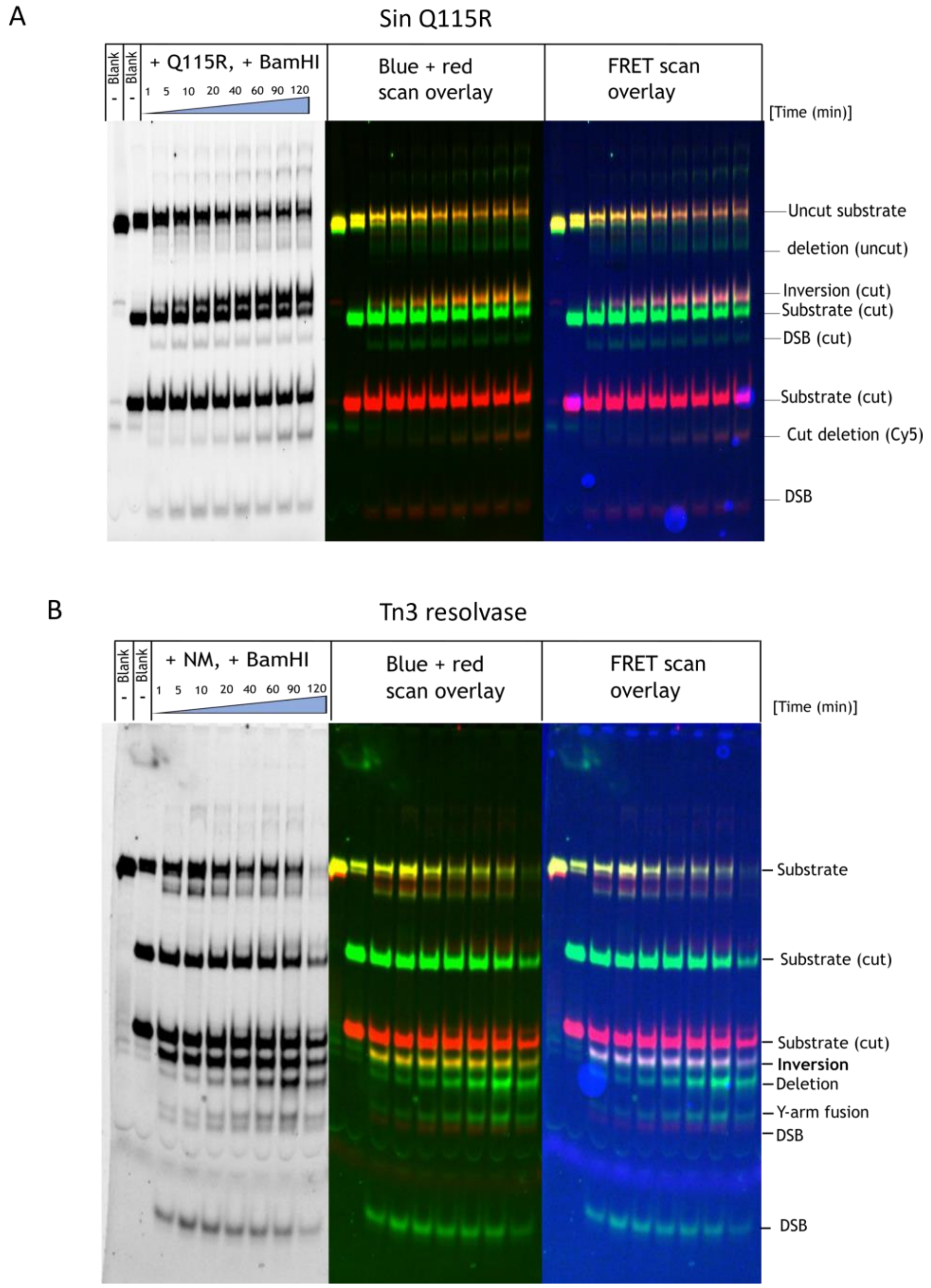

Supplementary figure 6: Time course assays analysed on 5% polyacrylamide gel containing 0.1% v/v SDS. An overlaid image showing fluorescence from both donor (green) and acceptor (red) scans is shown in colour and greyscale (used for band analysis). The FRET scan shows an overlay of the donor and acceptor emission scans (upon donor excitation). Fragments containing a yellow band in the FRET scan contain a donor and acceptor which are too far apart to exhibit FRET, and pink/ white fragments contain a pair exhibiting FRET (pink due to green/white overlap). **A.** SUL25/ Sin Q115R recombination. The gels shown are in good accordance with triplicate repeats of the recombination assays. **B.** UL25/ Tn3 NM recombination (see Methods for experimental details).

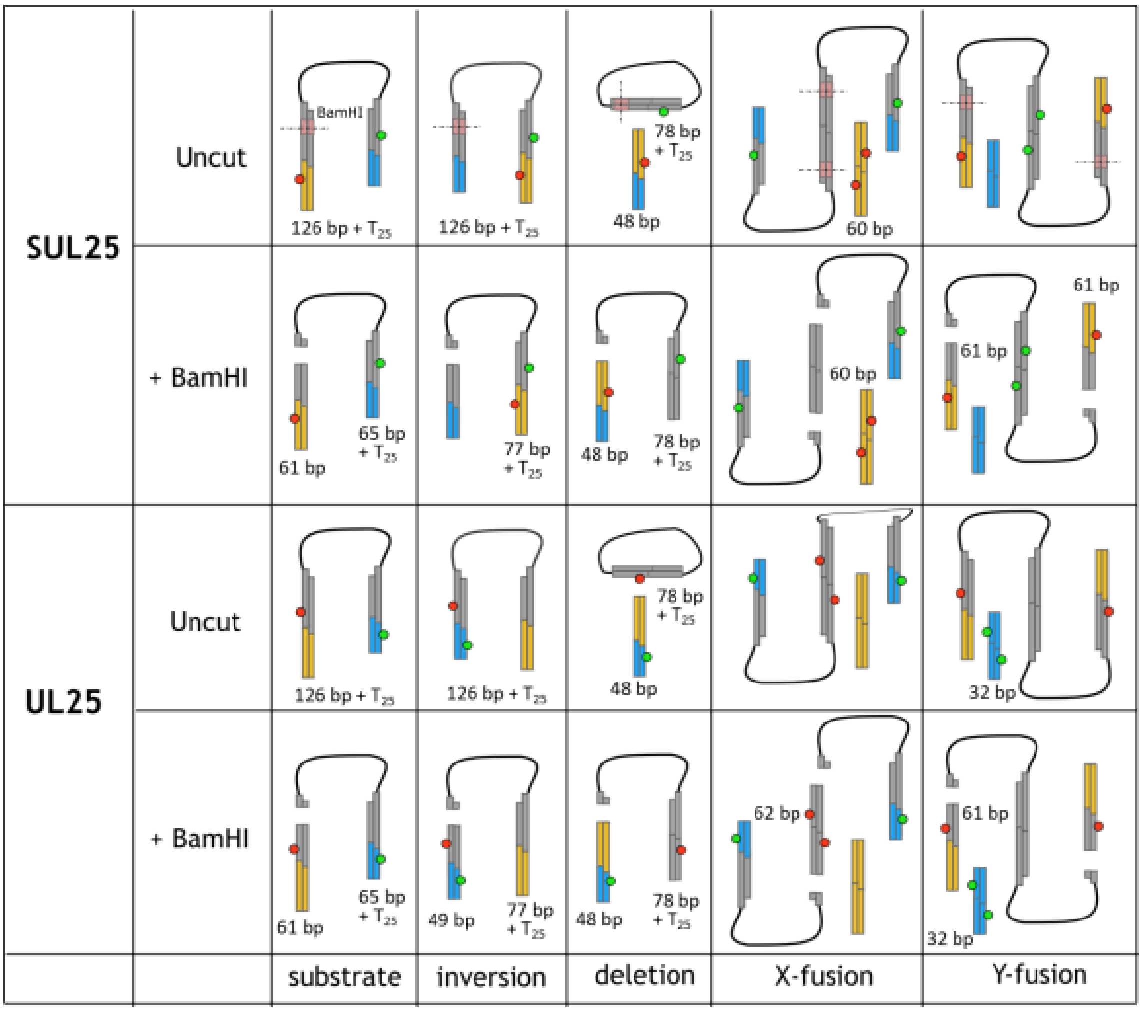

Supplementary figure 7: Products of potential recombination pathways using linked site I substrates SUL25 and UL25, and fragments from restriction digest by BamHI. The desired intramolecular recombination pathway occurs when site I pairs form an “inversion-ready” synapse (orange and blue halves on the same side as shown in the substrate cartoon) resulting in a single inversion product, bringing fluorescent dyes, Cy5 and Alexa 488 (or Atto 532) (shown as red and green circles respectively) closer, causing FRET. Synapsis in “deletion-ready” alignment results in ‘circularized’ and linear recombinant products, each with one fluorophore (hence no FRET). The lower row for each substrate shows the results of a restriction digest with BamHI endonuclease, creating characteristic fluorescent fragments for each reaction pathway which can be separated by SDS-PAGE. Sizes of all small fluorescent fragments are indicated. Intermolecular recombination can also occur, giving additional minor products (X-fusion and Y-fusion columns).

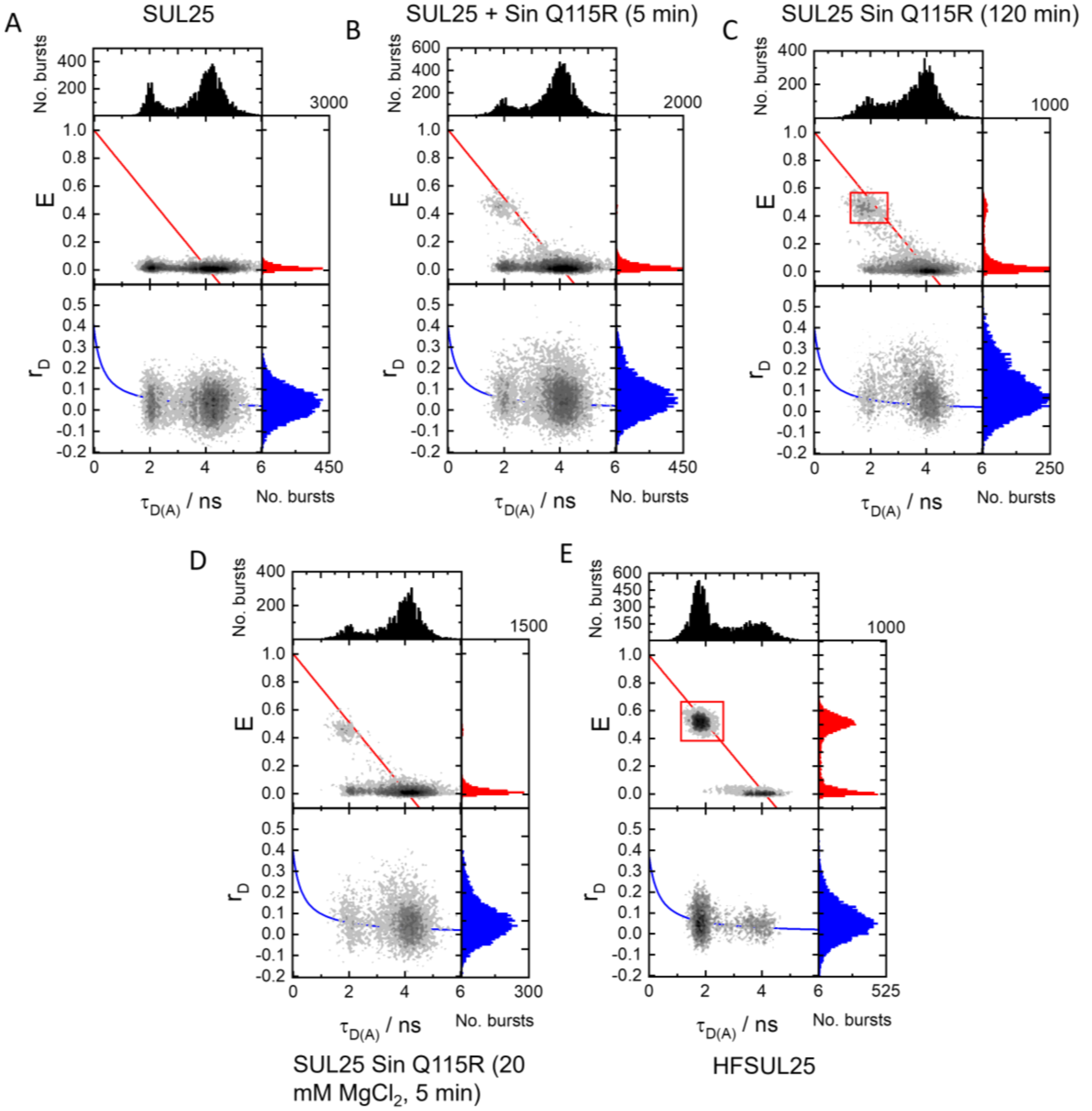

Supplementary figure 8: SM-FRET of linked site I substrate SUL25 in the presence and absence of Sin Q115R, analysed by MFD. 2D burst-frequency histograms of FRET efficiency (E) and donor anisotropy (r_D) plotted against donor lifetime in the presence of the acceptor ($\tau_{D(A)}$) are shown for all samples. **A.** SUL25 substrate without Sin Q115R has no detectable FRET as the inter-fluorophore distance is outside the range of FRET sensitivity. The zero-FRET population shows Alexa 488 τ = 4.0 ns and a quenched population with τ = 2.0 ns, which is commonly observed for Alexa 488 on DNA [2]. **B**. SUL25 was incubated with Sin Q115R for 5 minutes at 37 °C in the absence of $MgCl_2$. One FRET population corresponding to the inversion product is observed, at low concentration. **C.** The concentration of the inversion population increases, and the zero-FRET population is reduced after 120 minutes, suggesting that inversion progresses efficiently over the 120-minute reaction period. **D.** A similar population is observed after 5 minutes of recombination (as in B) when 20 mM $MgCl_2$ is added. **E.** Substrate HFSUL25, which is identical to the inversion product of SUL25, without Sin Q115R. The expected high E value is observed.

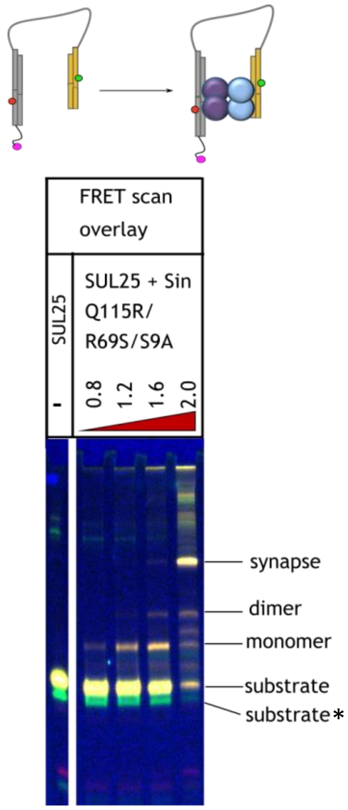

Supplementary figure 9: Binding of Sin Q115R/R69S/S9A to substrate SUL25. Numbers illustrate Sin concentration (given in μM). The protein was added to SUL25 on ice, then the samples were loaded onto a 5% polyacrylamide-TBE gel and separated by electrophoresis at at 4 °C. Alexa 488 (green) + Cy5 (red) FRET scan overlay is shown. Fragments that appeared in the FRET scan are coloured blue, such that when overlaid with the donor/ acceptor scans, fragments which exhibit FRET would appear as white/ pink (see Methods). Fragments which contain both donor and acceptor fluorophores but do not exhibit FRET appear yellow, as is observed for substrate, monomer, dimer, and also synapse in this case. The gel shown is representative of the similar results generated in three replicated experiments. *Shows substrate which is not fully annealed (loss of acceptor oligo).

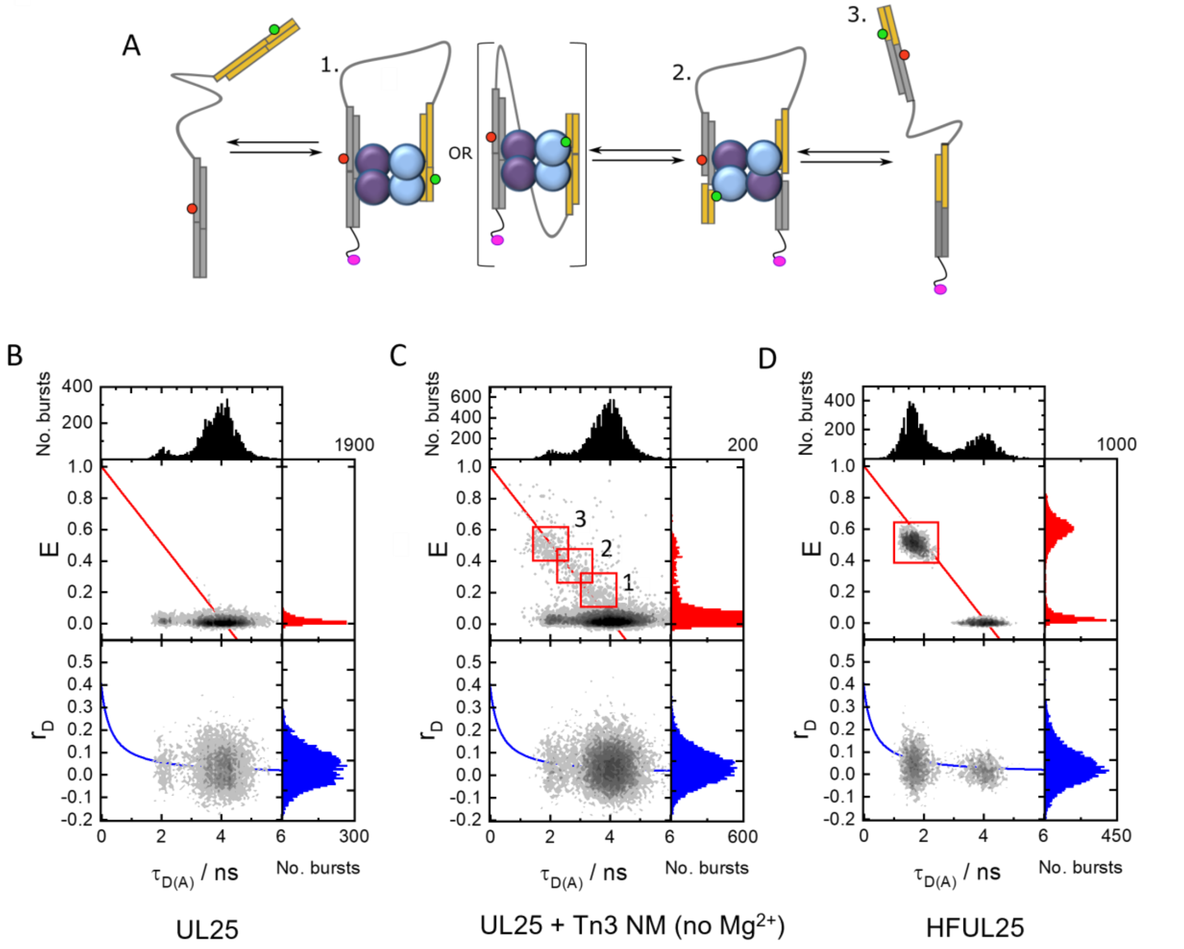

Supplementary figure 10: SM-FRET of linked site I substrate UL25 in the presence and absence of Tn3 NM resolvase, analysed by MFD: 2D burst-frequency histograms of FRET efficiency (E) and donor anisotropy (r_D) plotted against donor lifetime in the presence of the acceptor ($\tau_{D(A)}$) are shown for all samples. **A.** Reaction scheme showing fluorophore positions at each stage of recombination with UL25 and Tn3 NM resolvase. **B.** UL25 substrate alone has zero FRET, τ = 4.02 ns. **C.** UL25 incubated with Tn3 NM resolvase in the absence of $MgCl_2$. The recombination pathway results in three FRET states (red boxes), corresponding to (1) the synapse (in parallel OR antiparallel conformation) with a low E (E = 0.24 ± 0.01), (3) the inversion product with a high E (E = 0.52 ± 0.01), and (2) DNA-cleaved recombination intermediates which have a medium E value (E = 0.37 ± 0.01). **D.** Substrate HFUL25 is identical to the inversion product DNA molecule from UL25, and shows the expected high E value (E = 0.50 ± 0.06).

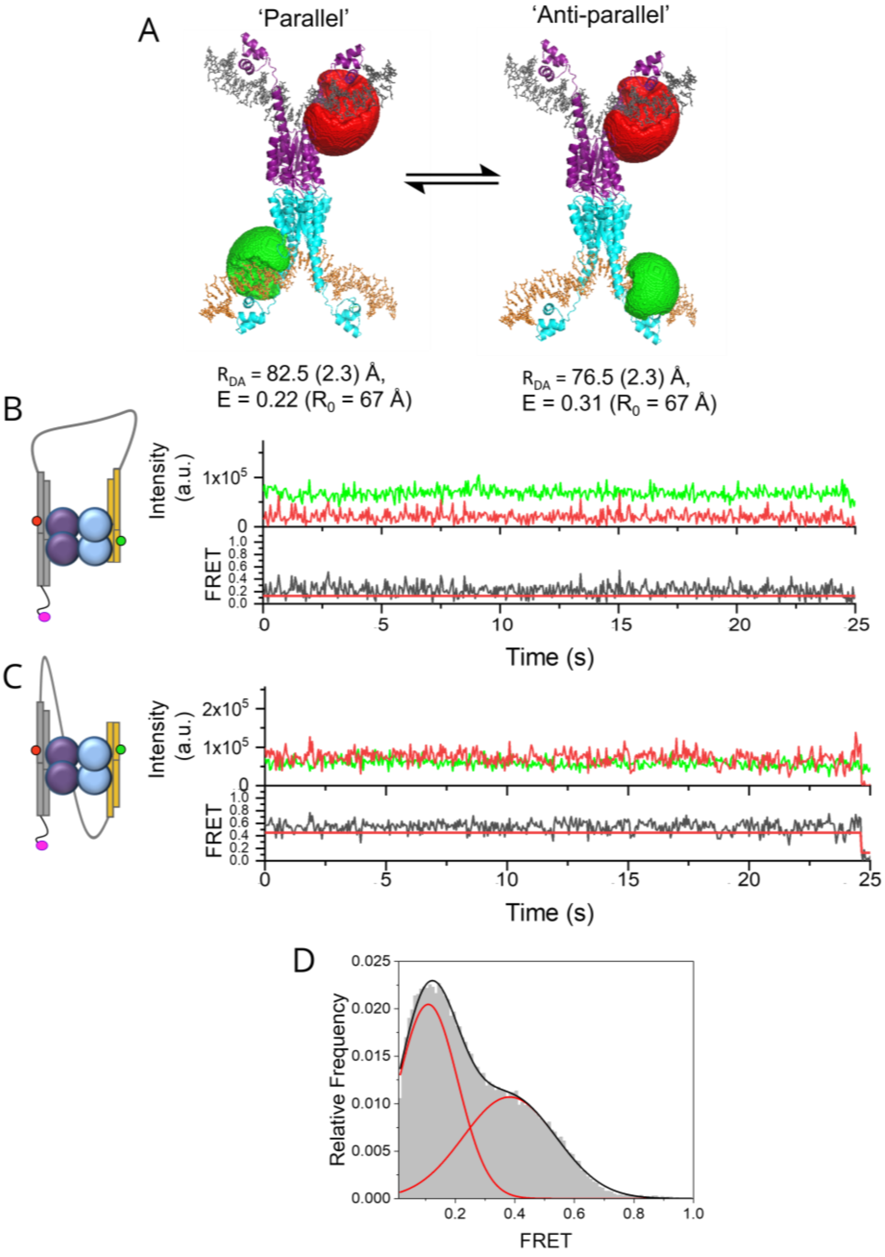

Supplementary figure 11: Parallel and antiparallel synapse alignments. A. AV simulations of the Cy3B/ Cy5 FRET pair positions in the UL25 synapse, using the structure for a $\gamma\delta$ resolvase dimer bound to site I DNA (pdb: 1GDT)[3] to simulate the FRET distances for the ligated non-recombinant structure. The parallel synapse (left) leads to the inversion pathway of recombination and is predicted to have a lower FRET efficiency. The antiparallel synapse (right) leads to the deletion pathway and is predicted to have a medium FRET efficiency. **B-C.** TIRF time trajectories of surface-immobilized UL25/ Tn3 NM/S10A (catalytically inactive mutant) synapses showing one possible 'parallel' synapse conformation (B) and one 'anti-parallel' synapse conformation (C). **D.** FRET population histogram of UL25 in the presence of Tn3 NM/S10A resolvase (195 molecules). The E values from Gaussian fits are E_{par} = 0.11 (0.01) and E_{anti} = 0.38 (0.01). Note that no traces displayed dynamic switching behaviour between these two conformations; all traces had a single, stable FRET value.

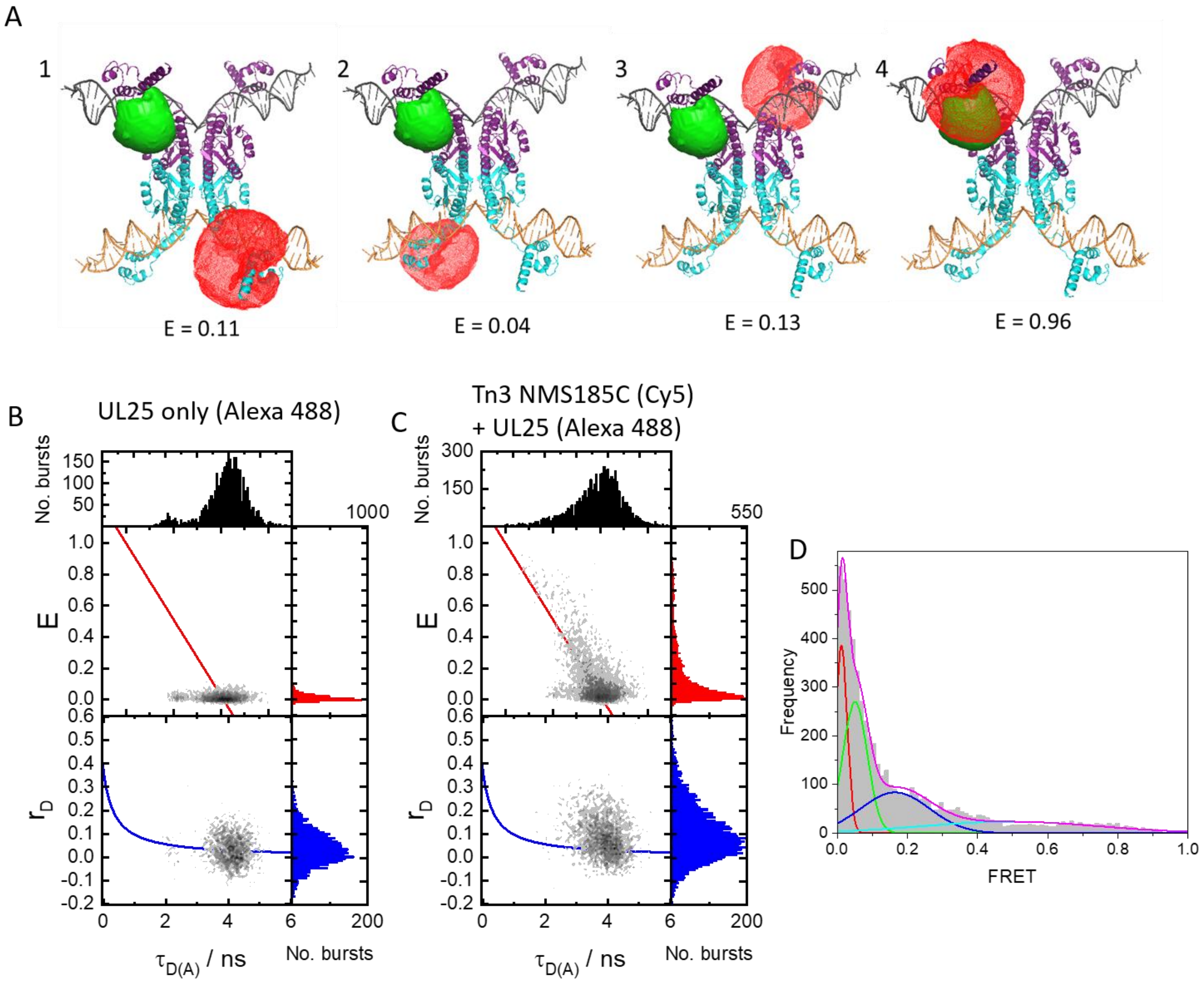

Supplementary figure 12: Accessible volume simulations and MFD studies of recombination of UL25 (Alexa 488-labelled) by Tn3 NM resolvase (Cy5-labelled). A. AV simulations showing four possible FRET-pair positions depending on where the fluorescent resolvase subunit is located within the synapse. The AV of the Alexa 488 donor fluorophore attached to the UL25 DNA is shown as a green surface sphere and the Cy5 acceptor fluorophore attached to resolvase at position 185 is shown as red mesh. An R_0 value of 51.8 Å for Alexa 488/ Cy5 was used in the analysis [4]. Synapses containing multiple fluorescent resolvase subunits are also possible; these could contribute additional FRET populations not specified in A. **B-C**. 2D burst-frequency histograms of FRET efficiency (E) and donor anisotropy (r_D) vs donor/ acceptor lifetime $\tau_{D(A)}$ are shown; **B,** without Tn3 NM (Cy5), and **C,** with Tn3 NM (Cy5). **D.** FRET efficiency histogram showing individual populations after Tn3 NM (Cy5) addition. The peaks best fit to 4 states (including the zero-FRET state), which are likely a combination of the possible FRET positions as shown in A.

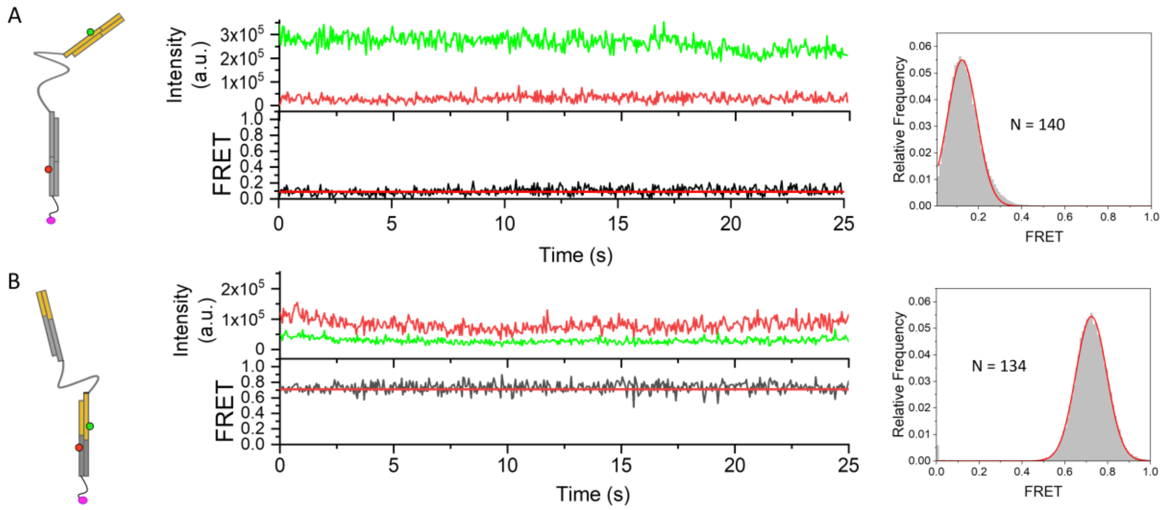

Supplementary figure 13: SmFRET trajectories of immobilized SUL25 and HFSUL25 substrates. Donor (green) and acceptor (red) intensity traces are shown in the top panels, and FRET traces (black) after Hidden Markov Modelling (HMM) fit (red) are shown in the bottom panels. (**A** and **B**) TIRF time trajectories of surface-immobilized SUL25 (**A**) and HFSUL25 (**B**), and FRET histograms of total saved traces (after filtering traces that showed photobleaching/blinking). The substrate cartoons on the left are as in Fig. 2; the magenta oval indicates the attachment point to the glass surface. Fluorophores in SUL25 are situated far apart (separate sites) but are close together in HFSUL25, showing the FRET efficiencies that would be expected before and after inversion.

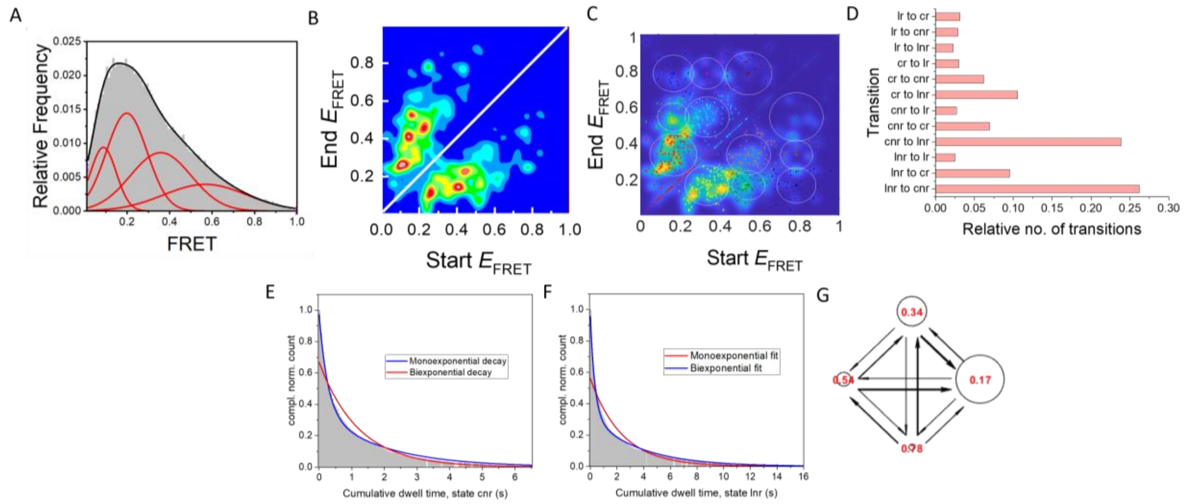

Supplementary figure 14: Modelling of the single-molecule FRET data for SUL25/Sin Q115R reaction at 21 °C. A. FRET population histogram of all dynamic traces. For SUL25 recombination at 21 °C (N = 136 molecules), the histogram fit to four populations with E = 0.08 (0.01), E = 0.20 (0.05) and E = 0.36 (0.28), and E = 0.57 (0.23) taken from a Gaussian fit. **B.** FRET population histogram of all dynamic traces. For SUL25 recombination (N = 132 molecules), the histogram shows four distinct populations with E = 0.09 (0.01), E = 0.20 (0.04) and E = 0.40 (0.18), and E = 0.70 (0.23) taken from a Gaussian fit. **C.** Gaussian convoluted transition density plot. **D**. A clustering algorithm is applied to determine the overall state configuration. The most sufficient cluster model is determined by the Bayesian Information Criterion (BIC), which has the lowest value when the number of states (J) = 4 [5]. **E.** Results from 4-state model inference; E = 0.17 (0.04), E = 0.34 (0.02), E = 0.54 (0.03), E = 0.78 (0.04), (see Table 1 in the main text for structural assignment). Plot of the relative number of transitions for all possible states is shown, obtained from inferred kinetic parameters. **F**. Dwell time histograms of subunit rotation transitions between states cnr and cr (See Fig. 3 in the main text for details on the structures of the states). Both histograms (shown in grey) are fitted to monoexponential and biexponential decay functions and dwell times were computed, with 180° rotation in the forward (cnr- cr) direction displayed on the left and in the backward (cr- cnr) direction on the right. **G.** Trellis diagram showing the most probable kinetic model with states depicted as circles (larger is a more frequented state) and transitions as arrows (larger is a more probable transition).

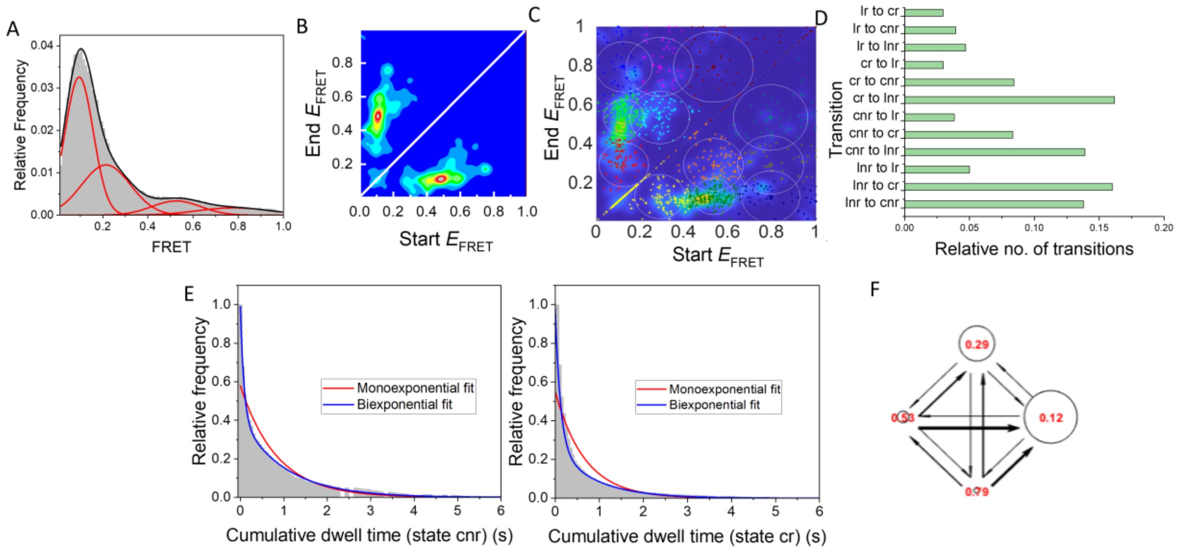

Supplementary figure 15: Modelling of the single-molecule FRET data for SUL25/Sin Q115R reaction in the presence of 20 mM $MgCl_2$ at 37 °C. **A.** FRET population histogram of all dynamic traces. For SUL25 recombination (N = 166 molecules), the histogram shows four distinct populations with E = 0.10 (0.01), E = 0.21 (0.11) and E = 0.53 (0.06), and E = 0.78 (0.33) taken from a Gaussian fit. **B.** Gaussian convoluted transition density plot. **C**. A clustering algorithm is applied to determine the overall state configuration. The most sufficient cluster model is determined by the Bayesian Information Criterion (BIC), which has the lowest value when the number of states (J) = 4 [5]. **D.** Results from 4-state model inference; E = 0.13 (0.04), E = 0.29 (0.03), E = 0.53 (0.03), E = 0.79 (0.04), (see Table 1 in the main text for structural assignment). Plot of the relative number of transitions for all possible states is shown, obtained from inferred kinetic parameters. **E**. Dwell time histograms of subunit rotation transitions between states cnr and cr (See Fig. 3 in the main text for details on the structures of the states). Both histograms (shown in grey) are fitted to monoexponential and biexponential decay functions and dwell times were computed, with 180° rotation in the forward (cnr- cr) direction displayed on the left and in the backward (cr- cnr) direction on the right. **F.** Trellis diagram showing the most probable kinetic model with states depicted as circles (larger is a more frequented state) and transitions as arrows (larger is a more probable transition).

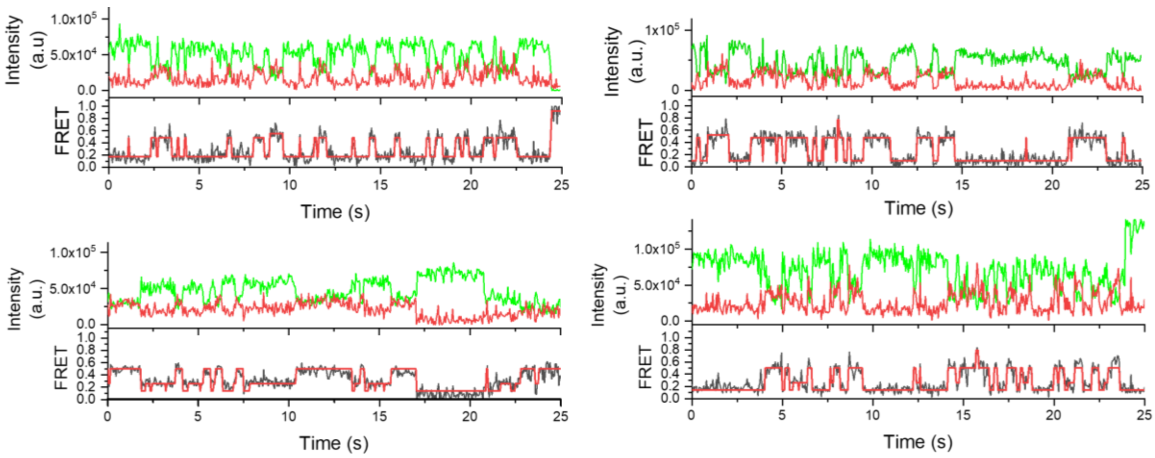

Supplementary figure 16: Single-molecule trajectories of immobilized SUL25 in the presence of Sin Q115R at 37 °C. Further examples of traces showing dynamic anticorrelation between states cnr and cr, highlighting that multiple rotations can occur during the 25 s recording time.

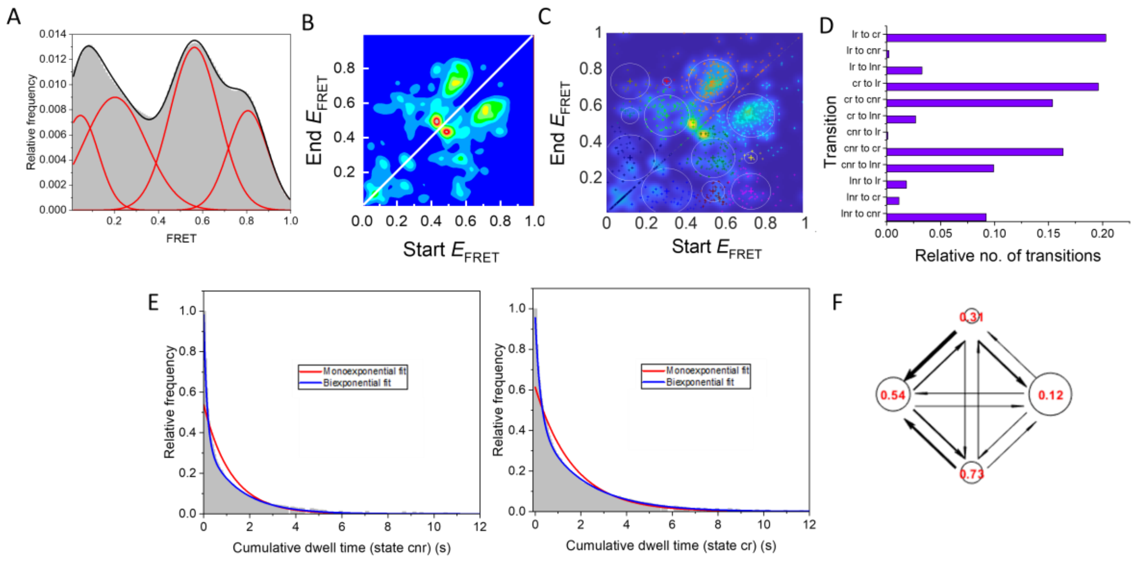

Supplementary figure 17: Modelling of the UL25/Tn3 NM resolvase single-molecule FRET data at 21 °C. **A.** FRET population histogram of all dynamic traces. For immobilized UL25 in the presence of Tn3 NM resolvase (**A**), (N = 163 molecules), the histogram shows four distinct populations with E = 0.07 (0.01), E = 0.20 (0.04), 0.48 (0.04) and 0.70 (0.04), taken from a Gaussian fit. **B.** Gaussian convoluted transition density plots. **C**. A clustering algorithm is applied to determine the overall state configuration. The most sufficient cluster model is determined by the Bayesian Information Criterion (BIC), which has the lowest value when the number of states (J) = 4. [5]. **D.** Results from 4-state model inference. E = 0.12 (0.03), E = 0.31 (0.03), E = 0.54 (0.03), E = 0.73 (0.02), (see Table 1 (main text) for structural assignment). A plot of the relative number of transitions for all possible states is shown, obtained from inferred kinetic parameters. **E**. Dwell time histograms of subunit rotation transitions between states cnr and cr (See Fig. 3 main text for details on structure of states). Both histograms (shown in grey) are fitted to single exponential decay functions and dwell times were computed, with 180° rotation from state cnr displayed on the left and from state cr direction on the right. **F.** Trellis diagram showing the most probable kinetic model with states depicted as circles (larger is a more frequented state) and transitions as arrows (larger is a more probable transition). Numbers are FRET efficiency values.

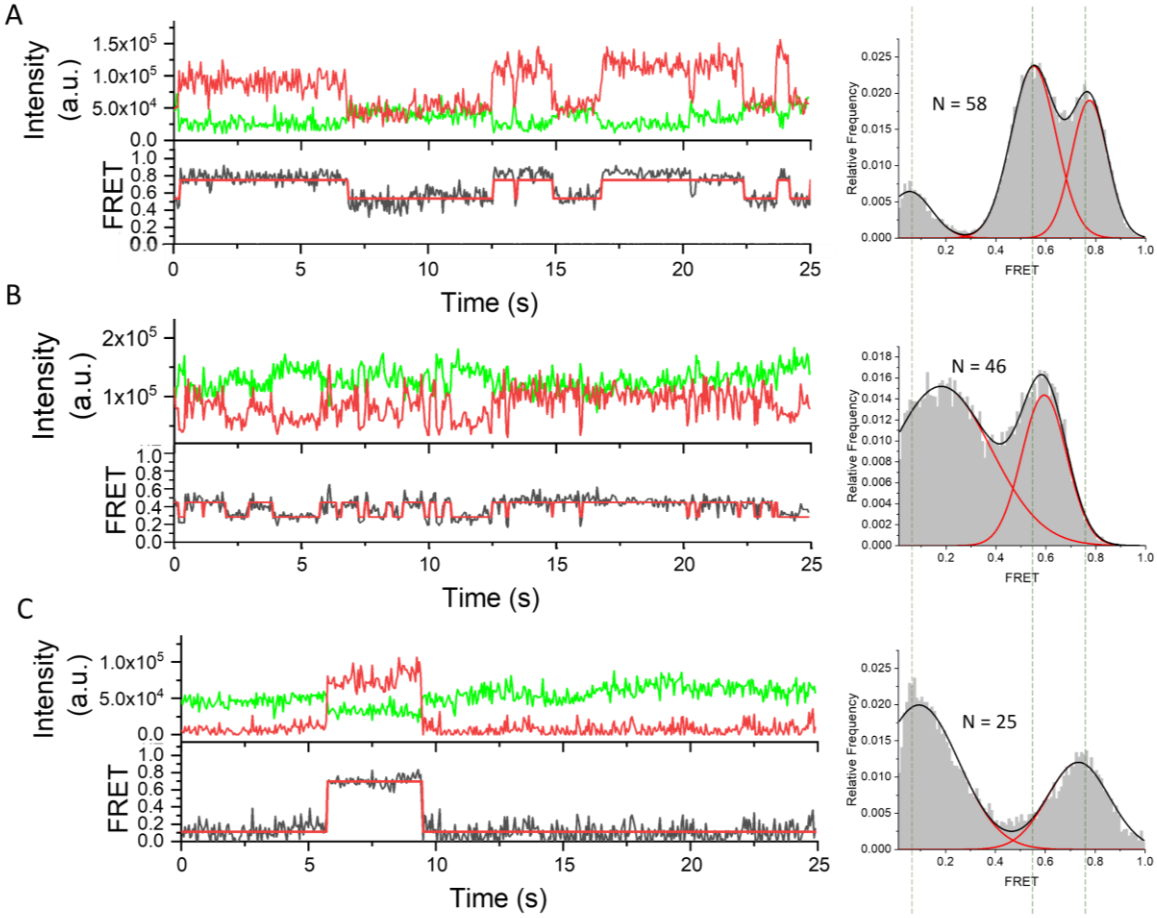

Supplementary figure 18: **Single-molecule trajectories of immobilized UL25 in the absence and presence of Tn3 NM resolvase.** Donor (green) and acceptor (red) intensity traces are shown in the top panels, and FRET traces (black) with FRET after HMM fit (red) are shown in the bottom panels. **A**. Reversible transitions between state 3 (E = 0.54 (0.03)) and state 4 (E = 0.73 (0.03)) are proposed to correspond to the conformational change from cleaved recombinant synapse to the ligated recombinant product. **B**. Reversible transitions between state 2 (E = 0.31 (0.03)) and state 3 are proposed to correspond to the conformational change between cleaved non-recombinant and cleaved recombinant. The data are consistent with a subunit rotation mechanism. **C**. Reversible transitions between state 1 (E = 0.12 (0.03)) and 4 were also observed.

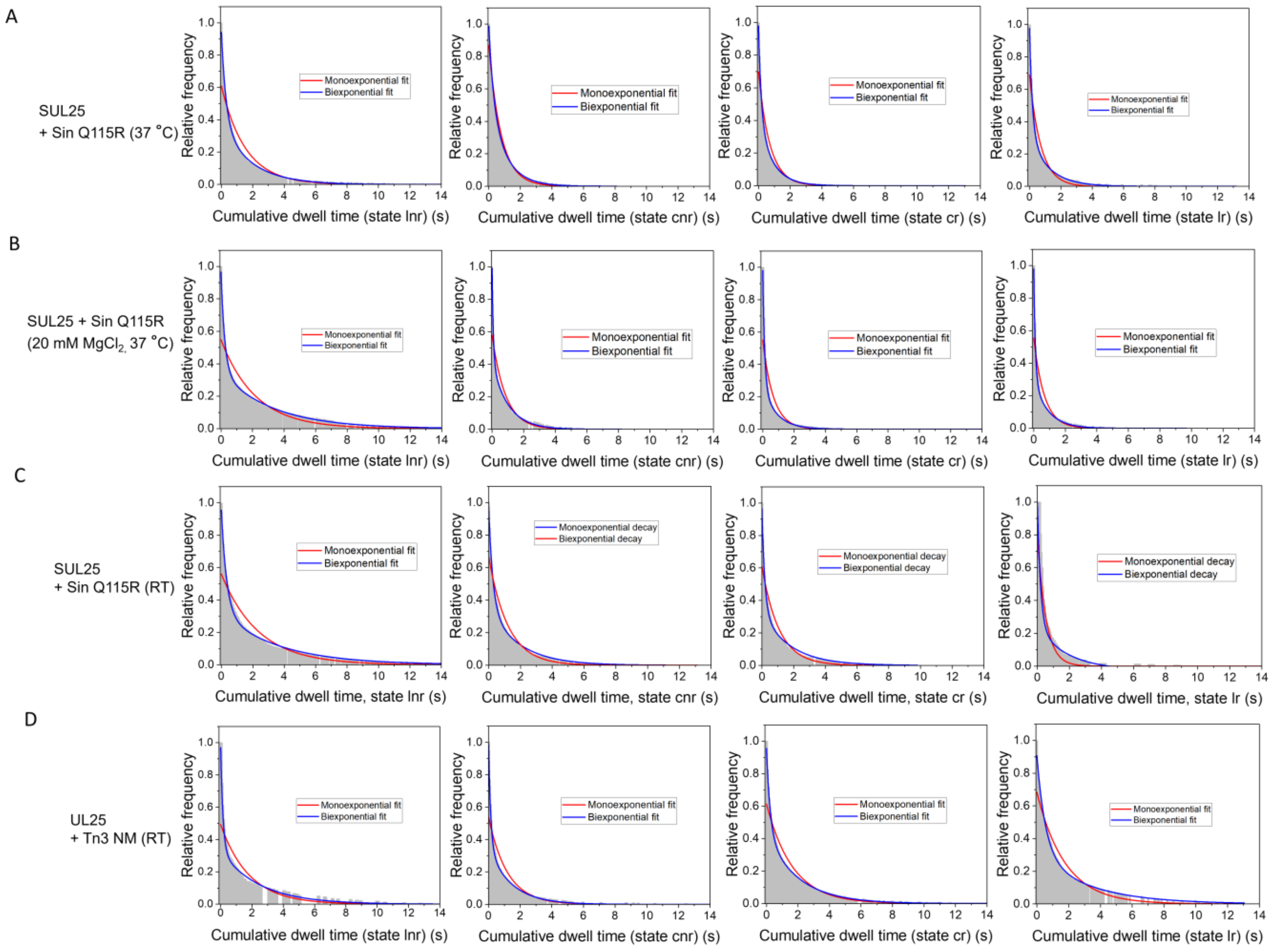

Supplementary figure 19: Comparison of single and biexponential decays fitted to cumulative dwell time histograms obtained from TIRF analysis of recombination. Fits of each state obtained from TIRF analysis of recombination with Sin Q115R in the absence (**A**) and presence of Mg^{2+} at 37 °C (**B**), and at room temperature in the absence of Mg^{2+} (**C**). Recombination usingTn3 NM at room temperature, in the absence of Mg^{2+} is also shown in (**D**). For further details on the effect of Mg^{2+} on recombination see “Recombination of freely diffusing complexes” section in the main text.

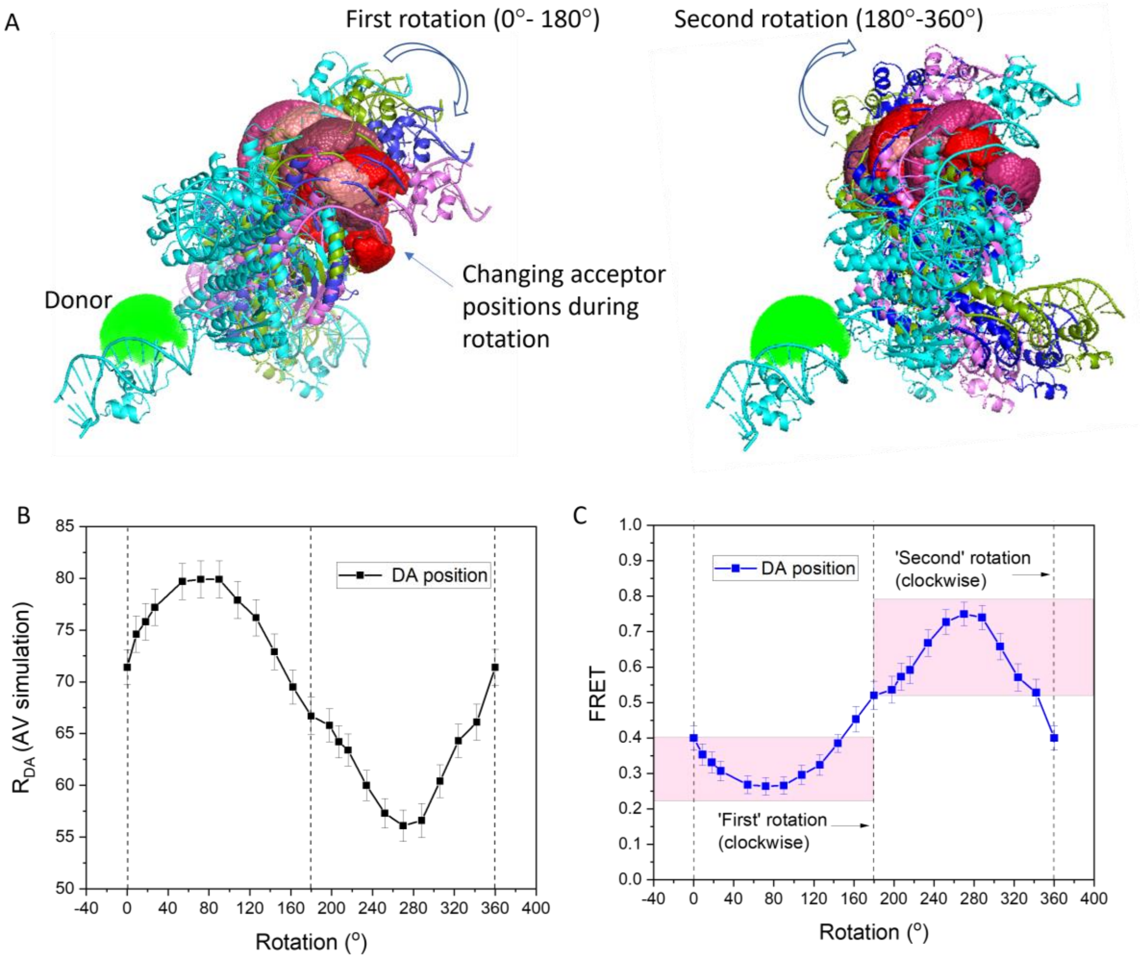

Supplementary figure 20: Predicted donor/ acceptor distances and corresponding E values for each rotational state. Rotational states were simulated using the γδ resolvase-DNA co-crystal structure (pdb: 1ZR4[6]) and AV simulations were used to predict FRET distances. At 0°, the FRET positions are the same as in the cnr state for UL25 modelled onto 1ZR4 (Fig. 3, main text). **A.** Snapshot PyMOL images of γδ-resolvase crystal structure (pdb: 1ZR4) at various angles of rotation are shown in different colours, along with the changing position of the acceptor, shown in different shades of red. Due to the position of the donor (shown in green) on the stationary half of the tetramer, on the 'first rotation' (0°-180°) the donor/ acceptor distance begins large and decreases, and on the 'second rotation' (180°-360°) the donor/ acceptor distance begins small and increases. **B.** R_{DA} distances predicted for steps of a complete 360° rotation using UL25 fluorophore positions. **C.** E values predicted for steps of a complete 360° rotation using UL25 fluorophore positions. The pink boxes illustrate the difference in expected FRET values for the 'forward' (0°- 180°) compared with 'reverse' (180°- 360°) rotations.

References

1. Koenig SLB, Hadzic M, Fiorini E, Boerner R, Kowerko D, Blanckenhorn WU, Sigel RKO. BOBA FRET: Bootstrap-Based Analysis of Single-Molecule FRET Data. *Plos One* **8**, (2013).

2. Dey SK, Pettersson JR, Topacio AZ, Das SR, Peteanu LA. Eliminating Spurious Zero-Efficiency FRET States in Diffusion-Based Single-Molecule Confocal Microscopy. *J. Phys. Chem. Lett.* **9**, 2259-2265 (2018).

3. Yang W, Steitz TA. Crystal-structure of the site-specific recombinase Gamma-delta resolvase complexed with a 34 bp cleavage site. *Cell* **82**, 193-207 (1995).

4. Wozniak AK, Schroeder GF, Grubmueller H, Seidel CAM, Oesterhelt F. Single-molecule FRET measures bends and kinks in DNA. *Proc. Natl Acad. Sci. USA* **105**, 18337-18342 (2008).

5. Hadzic MCAS, Boerner R, Koenig SLB, Kowerko D, Sigel RKO. Reliable State Identification and State Transition Detection in Fluorescence Intensity-Based Single-Molecule Forster Resonance Energy-Transfer Data. *J. Phys. Chem. B.* **122**, 6134-6147 (2018).

6. Li WK, Kamtekar S, Xiong Y, Sarkis GJ, Grindley NDF, Steitz TA. Structure of a synaptic gamma delta resolvase tetramer covalently linked to two cleaved DNAs. *Science* **309**, 1210-1215 (2005).
