# Supplementary material for: Direct observation of subunit rotation during DNA strand exchange by serine recombinases
Source: Nat Commun. 2024 Nov 29;15:10407. doi: 10.1038/s41467-024-54531-4 (PMC11607074; doi:10.1038/s41467-024-54531-4)
Supplement: Supplementary file 2 — Reporting Summary [file 41467_2024_54531_MOESM2_ESM.pdf]

nature portfolio

Corresponding author(s): Gillian M. Cadden, Jan-Gero Schloetel, Grant McKenzie, Martin R. Boocock, Steven W. Magennis, W. Marshall Stark

Last updated by author(s): Oct 11, 2024

Reporting Summary

Nature Portfolio wishes to improve the reproducibility of the work that we publish. This form provides structure for consistency and transparency in reporting. For further information on Nature Portfolio policies, see our Editorial Policies and the Editorial Policy Checklist.

Statistics

For all statistical analyses, confirm that the following items are present in the figure legend, table legend, main text, or Methods section.

n/a	Confirmed	
☐	☒	The exact sample size (*n*) for each experimental group/condition, given as a discrete number and unit of measurement
☒	☐	A statement on whether measurements were taken from distinct samples or whether the same sample was measured repeatedly
☒	☐	The statistical test(s) used AND whether they are one- or two-sided<br>*Only common tests should be described solely by name; describe more complex techniques in the Methods section.*
☒	☐	A description of all covariates tested
☒	☐	A description of any assumptions or corrections, such as tests of normality and adjustment for multiple comparisons
☐	☒	A full description of the statistical parameters including central tendency (e.g. means) or other basic estimates (e.g. regression coefficient) AND variation (e.g. standard deviation) or associated estimates of uncertainty (e.g. confidence intervals)
☒	☐	For null hypothesis testing, the test statistic (e.g. *F*, *t*, *r*) with confidence intervals, effect sizes, degrees of freedom and *P* value noted<br>*Give P values as exact values whenever suitable.*
☒	☐	For Bayesian analysis, information on the choice of priors and Markov chain Monte Carlo settings
☒	☐	For hierarchical and complex designs, identification of the appropriate level for tests and full reporting of outcomes
☒	☐	Estimates of effect sizes (e.g. Cohen's *d*, Pearson's *r*), indicating how they were calculated

Our web collection on statistics for biologists contains articles on many of the points above.

Software and code

Policy information about availability of computer code

Data collection	MFD- Data collection used software written by the group of Prof. Claus Seidel (Heinrich Heine Universität, Düsseldorf).<br>TIRF- .tif movies were recorded using Image Pro-Plus 7.0 software.<br>AV simulations- completed using freely available software FPS version 1.1. AV cloud images were generated using PyMOL.
Data analysis	Fluorescent gel band analysis- Fluorescence intensities of the bands were determined using ImageQuant 10.2.<br>MFD- Data analysis used software written by the group of Prof. Claus Seidel (Heinrich Heine Universität, Düsseldorf).<br>TIRF- tif movies were analysed using a commercially available program called TwoTone 3.1. Fluorescence vs. time trajectories were visualized and analysed using MATLAB procedures written in-house. All dynamic trajectories were analyzed individually without stitching using software packages 'HaMMy' and MASH-FRET' version 1.3.2.

For manuscripts utilizing custom algorithms or software that are central to the research but not yet described in published literature, software must be made available to editors and reviewers. We strongly encourage code deposition in a community repository (e.g. GitHub). See the Nature Portfolio guidelines for submitting code & software for further information.

Data

Policy information about availability of data

All manuscripts must include a data availability statement. This statement should provide the following information, where applicable:

- Accession codes, unique identifiers, or web links for publicly available datasets
- A description of any restrictions on data availability
- For clinical datasets or third party data, please ensure that the statement adheres to our policy

The data that underpin this work are available at : https://doi.org/10.5525/gla.researchdata.1641

Research involving human participants, their data, or biological material

Policy information about studies with human participants or human data. See also policy information about sex, gender (identity/presentation), and sexual orientation and race, ethnicity and racism.

Reporting on sex and gender	Human data was not used in this study.
Reporting on race, ethnicity, or other socially relevant groupings	Human data was not used in this study.
Population characteristics	Human data was not used in this study.
Recruitment	Human data was not used in this study.
Ethics oversight	Human data was not used in this study.

Note that full information on the approval of the study protocol must also be provided in the manuscript.

Field-specific reporting

Please select the one below that is the best fit for your research. If you are not sure, read the appropriate sections before making your selection.

☒ Life sciences ☐ Behavioural & social sciences ☐ Ecological, evolutionary & environmental sciences

For a reference copy of the document with all sections, see nature.com/documents/nr-reporting-summary-flat.pdf

Life sciences study design

All studies must disclose on these points even when the disclosure is negative.

Sample size	No less than 100 saved fluorescence vs. time trajectories that were used for further global analysis using HaMMy and MASH-FRET, though statistical analysis undertaken by McKinney et al. has shown that as few as 20 traces can be used with minimal statistical contribution to error.
Data exclusions	Fluorescence vs. time traces were excluded from analysis under the following circumstances:<br>1. Artifacts were observed due to blinking.<br>2. The fluorophores were subject to early photobleaching.<br>3. The signal-to-noise ratio was poor.<br>4. The total intensity (sum of the donor and acceptor intensities) showed continual changes possibly indicating nonspecific binding.
Replication	All experiments were repeated at least three times and showed consistent results.
Randomization	All measurements were independent of each other. Instruments settings were checked each day using appropriate controls.
Blinding	Blinding was not necessary as no inherent experimental bias was identified.

Reporting for specific materials, systems and methods

We require information from authors about some types of materials, experimental systems and methods used in many studies. Here, indicate whether each material, system or method listed is relevant to your study. If you are not sure if a list item applies to your research, read the appropriate section before selecting a response.

Materials & experimental systems

n/a	Involved in the study
☒	☐ Antibodies
☒	☐ Eukaryotic cell lines
☒	☐ Palaeontology and archaeology
☒	☐ Animals and other organisms
☒	☐ Clinical data
☒	☐ Dual use research of concern
☒	☐ Plants

Methods

n/a	Involved in the study
☒	☐ ChIP-seq
☒	☐ Flow cytometry
☒	☐ MRI-based neuroimaging

Plants

Seed stocks	Plants were not used in this study.
Novel plant genotypes	Plants were not used in this study.
Authentication	Plants were not used in this study.
