# Supplementary figures and images for: Direct observation of subunit rotation during DNA strand exchange by serine recombinases
Source: Nat Commun. 2024 Nov 29;15:10407. doi: 10.1038/s41467-024-54531-4 (PMC11607074; doi:10.1038/s41467-024-54531-4)

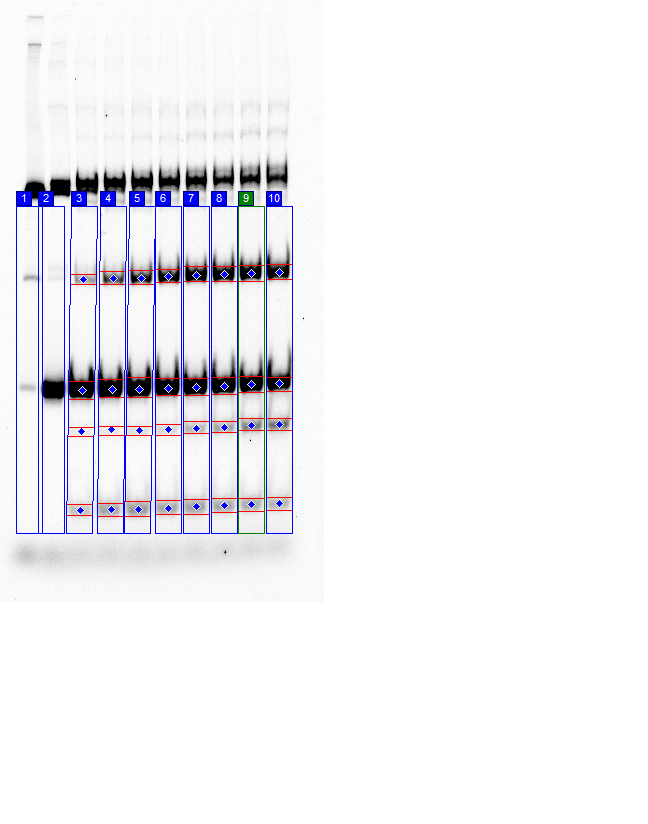

Supplement: Supplementary file 4 — Source Data [file 41467_2024_54531_MOESM4_ESM.zip › Source Data/Gel_Tn3NM_acceptorscan_fig4A.png]

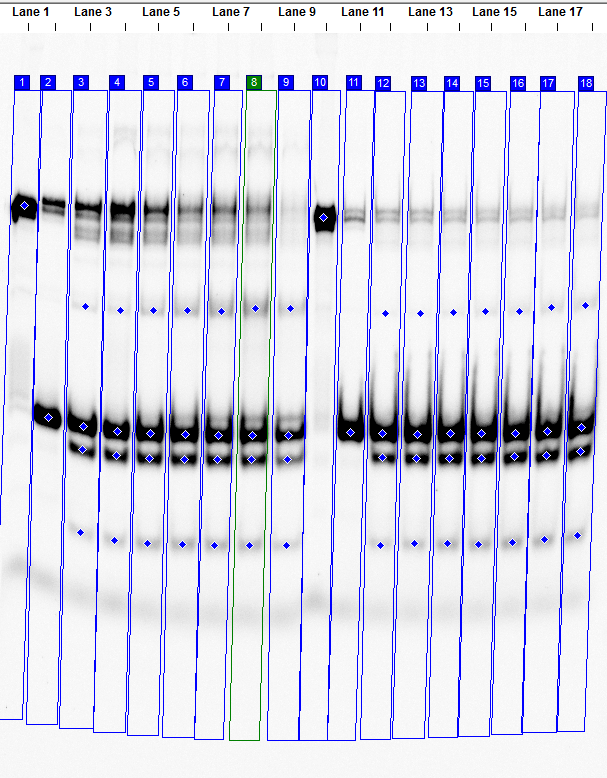

Supplement: Supplementary file 4 — Source Data [file 41467_2024_54531_MOESM4_ESM.zip › Source Data/Gel_Tn3NM_acceptorscan_fig4E.PNG]

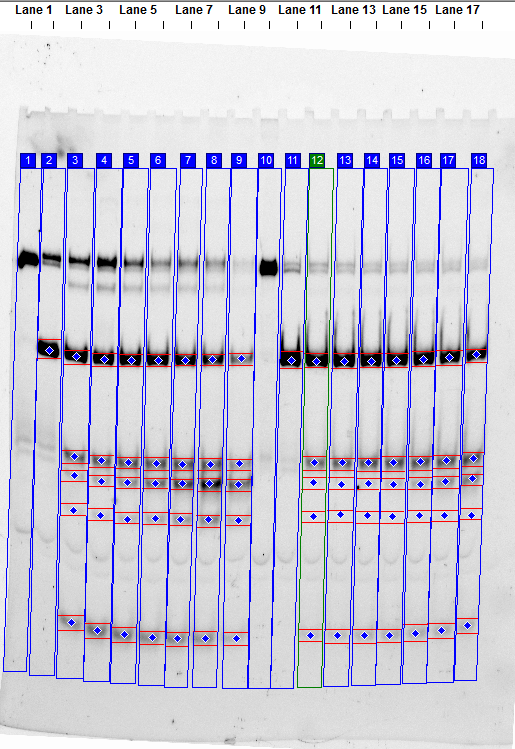

Supplement: Supplementary file 4 — Source Data [file 41467_2024_54531_MOESM4_ESM.zip › Source Data/Gel_Tn3NM_donorscan_fig4E.PNG]

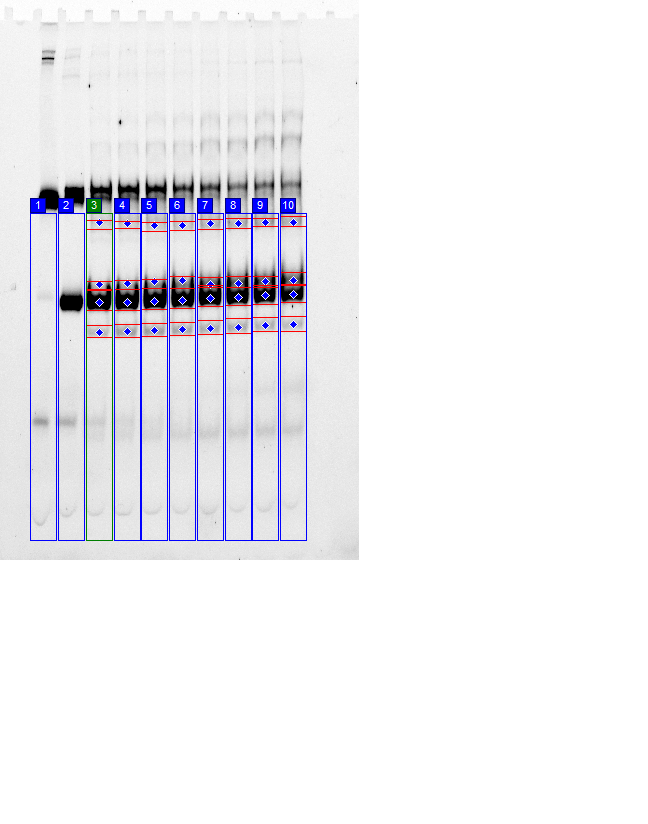

Supplement: Supplementary file 4 — Source Data [file 41467_2024_54531_MOESM4_ESM.zip › Source Data/Gel_Tn3NM_donorscan_fig4A.png]
